# Phenolic Constituents, Photoprotective Effect, and Antioxidant Capacities of *Achillea ligustica* All

**DOI:** 10.3390/molecules29174112

**Published:** 2024-08-30

**Authors:** Azza Bouteche, Ahmed Touil, Salah Akkal, Chawki Bensouici, Gema Nieto

**Affiliations:** 1 Laboratory of Natural Product from Plants and Organic Synthesis, Department of Chemistry, University of Mentouri Constantine 1, Constantine 25000, Algeria; azza.bouteche@umc.edu.dz (A.B.); ahmedtouil@yahoo.fr (A.T.); 2 Valorization of Natural Resources, Bioactive Molecules and Biological Analysis Unit, Department of Chemistry, University of Mentouri Constantine 1, Constantine 25000, Algeria; 3 National Center of Biotechnology Research, Constantine 25000, Algeria; chawkiislam@yahoo.fr; 4 Department of Food Technology, Nutrition and Food Science, Veterinary Faculty, University of Murcia, Regional Campus of International Excellence “Campus Mare Nostrum”, Campus de Espinardo, 30100 Murcia, Spain

**Keywords:** *Achillea ligustica*, flavonoids, total phenolic content, antioxidant tests, photoprotective effect

## Abstract

The present investigation was performed to figure out the chemical constituents and biological potential of polar extracts (AcOEt and BuOH) from *Achillea ligustica*, a medicinal species of the Asteraceae family. Liquid chromatography quadrupole time-of-flight mass spectrometry (LC-Q-TOF-MS) was utilized to conduct a preliminary analysis of the phytochemical profiles of the AcOEt and BuOH extracts. The analysis revealed the existence of twenty compounds in the AcOEt extract and twenty-two in the BuOH extract, classified into various types of secondary metabolites. Subsequently, the exudate from the plant yielded five flavonoids, including two 6-methoxyflavonols identified for the first time in this genus. The isolation of compounds from AcOEt and BuOH extracts was achieved through the combined use of column chromatography (silica gel and Sephadex LH-20) and preparative TLC chromatography. The structures have been elucidated using 1D and 2D NMR spectroscopy, alongside comparisons with research data. Our study measured the total phenolic and flavonoid contents and carried out a comprehensive range of antioxidant tests using DPPH, GOR, CUPRAC, reducing power, and *O*-phenanthroline assays. Both extracts exhibited considerable antioxidant potential and contained high levels of phenolic and flavonoid compounds. The photoprotective effect of the AcOEt and BuOH extracts was evaluated in vitro by measuring the sun protection factor. Both extracts exhibited a high capacity for UV radiation absorption. Consequently, this plant presents an intriguing prospect for future research focused on incorporating it into photoprotective cosmetic products and pharmaceutical formulations.

## 1. Introduction

The *Achillea* genus, belonging to the Asteraceae family, includes around 120 species predominantly found in Central Southwestern Asia and Southeastern Europe, with a distribution that extends across North America and Eurasia [1,2]. Five species have been identified within the Algerian flora, including *Achillea ligustica* All. [3]. *A. ligustica* is a plant with an aromatic scent and bitter taste; it can reach 100 cm in height. The species grows spontaneously in the Mediterranean region; in Algeria, it is called *Benkisson* [1,3].

In traditional medicine, *A. ligustica* has been employed to treat various ailments, including gastric pains, skin illnesses [4], headaches, cold pains, and rheumatic diseases [5], as well as healing battle wounds and stopping hemorrhages [6]. A range of pharmacological properties have been reported for this plant, such as antioxidant [7,8,9,10,11,12], antimicrobial [9,13,14], antifungal [15], neurotrophic [16], anti-psoriasis [12], antiproliferative, cytotoxicity and protective effects [10], and α-amylase inhibitory activity [8].

Several studies investigating the phytochemical profile of the *A. ligustica* species have revealed a diverse array of sesquiterpene lactones, with guaianolides being the predominant class. Notably, matricarin and desacetylmatricarin are among the most prevalent compounds. The plant is also characterized by a significant presence of flavonoids, including santin, 6-hydroxykaempferol 3,6-dimethyl, luteolin, apigenin, and apigenin 7-*O*-glucoside. The most frequently isolated monoterpene is (3RS,6RS)-2,6-dimethyl-1,7-octadiene-3,6-diol. In addition, the species has yielded two phenolic acids (caffeic acid and chlorogenic acid), along with one piperidine alkaloid, (2E,4E,6E,10E)-1-(1-piperidinyl)-2,4,6,10-tetradecatetraen-8-yn-1-one, and two non-polar compounds (β-sitosterol and 1-tricontanol) [1,8,10,16,17,18,19,20,21].

Sunlight exposure is crucial for human health in various ways, such as contributing to melanin production and aiding in the treatment of skin diseases such as psoriasis and vitiligo [22]. Nevertheless, extended exposure to solar ultraviolet (UV) radiation can lead to increased oxidative stress in skin cells, potentially initiating and promoting cancer [23]. Using sunscreen helps mitigate the risk of skin cancer associated with solar radiation [22]. Additionally, natural substances have been explored as potential sunscreen alternatives due to their ability to absorb UV radiation and their antioxidant properties [23], offering new approaches for preventing and treating UV-induced conditions [24].

Based on its diversity of secondary metabolites and its significant biological and pharmacological potential, the current study intended to define the chemical constituents of polar extracts of *A. ligustica* aerial parts collected in Algeria. Moreover, the study assessed the antioxidant and photoprotective properties through in vitro assays, along with quantifying the total phenolic and flavonoid contents.

## 2. Results

### 2.1. HPLC-Q-TOF-MS

The LC-Q-TOF-MS analysis of *A. ligustica* unveiled the existence of twenty compounds in the AcOEt extract and twenty-two in the BuOH extract, with retention times ranging from 3.22 to 26.31 min. The molecular formula, *m*/*z*, and the retention time (Rt) for each compound are listed in Table 1.

### 2.2. Structure of the Isolated Compounds

**Figure 1:**
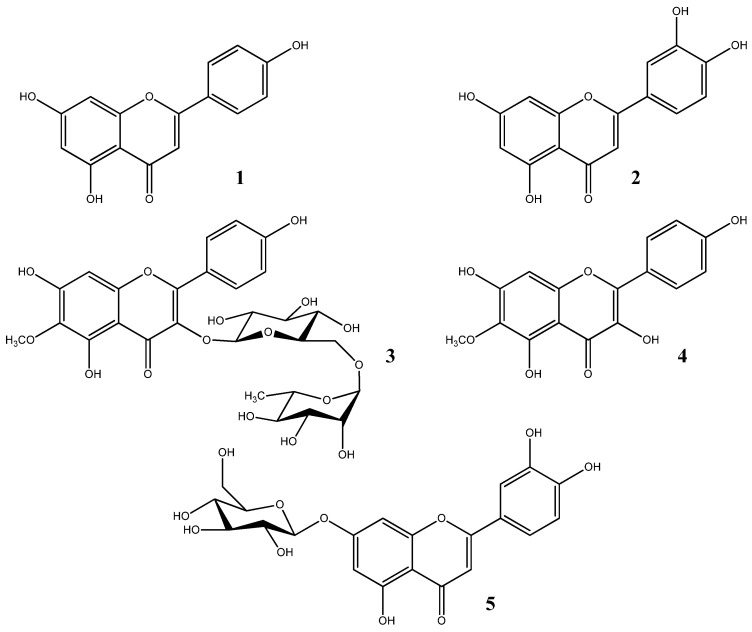
Compounds isolated from the AcOEt and BuOH extracts of *A. ligustica*.

The phytochemical investigation of the AcOEt and BuOH extracts obtained from the aerial parts of *A. ligustica* yielded five flavonoids, in which compounds (**3**) and (**4**) were isolated from the *Achillea* genus for the first time. The compounds’ structures were established through ^1^H-NMR, ^13^C-NMR, COSY, HSQC, and HMBC techniques alongside comparisons with research data. The structures of the isolated compounds are displayed in Figure 1, and the NMR spectrum data are provided in the Supplementary Materials.

The compounds were identified as apigenin (**1**) [25], luteolin (**2**) [26], 6-methoxykaempferol-3-*O*-rutinoside (**3**) [27], 6-methoxykaempferol (**4**) [28], and luteoline-7-*O*-β-glucoside (**5**) [29].

Apigenin (**1**), Yellow amorphous powder, ^1^H-NMR (400 MHz; DMSO-*d*_6_, *δ*(ppm), *J*(Hz)) 12.90 (1H, s, OH-5), 7.90 (2H, d, *J* = 8.8, H-2′/H-6′), 6.91 (2H, d, *J* = 8.8, H-3′/H-5′), 6.73 (1H, s, 3-H), 6.48 (1H, d, *J* = 2.1, H-8), 6.18 (1H, d, *J* = 2.1, H-6). ^13^C-NMR (100 MHz; CDCl_3_, *δ*(ppm)) 181.8 (C-4), 164.2 (C-7), 163.9 (C-2), 161.4 (C-4’), 161.2 (C-5), 157.4 (C-9), 128.5 (C-2′/C-6′), 121.2 (C-1′), 116.1 (C-3′/C-5′), 103.7 (C-10), 102.8 (C-3), 98.9 (C-6), 94.1 (C-8).

Luteolin (**2**), Yellow amorphous powder, ^1^H-NMR (400 MHz; DMSO-*d*_6_, *δ*(ppm), *J*(Hz)) 12.91 (1H, s, OH-5), 7.39 (1H, dd, *J* = 2.3–8.3, H-6′), 7.37 (1H, d, *J* = 2.2, H-2′), 6.88 (1H, d, *J* = 8.3, H-5′), 6.64 (1H, s, 3-H), 6.44 (1H, d, *J* = 2.1, H-8), 6.17 (1H, d, *J* = 2.1, H-6). ^13^C-NMR (100 MHz; CDCl_3_, *δ*(ppm)) 181.9 (C-4), 166.9 (C-2), 164.5 (C-7),164.1 (C-5), 161.6 (C-9), 157.5 (C-4′), 146.0 (C-3′), 121.6 (C-1′), 119.2 (C-6′), 116.3 (C-5′), 113.4 (C-2′), 103.8 (C-10), 103.0 (C-3), 99.1 (C-6), 94.2 (C-8). 

6-Methoxykaempferol-3-*O*-rutinoside (**3**) Yellow amorphous powder, ^1^H-NMR (600 MHz; CD_3_OD, *δ*(ppm), *J*(Hz)) 8.07 (2H, d, *J* = 8.6, H-2′/H-6′), 6.90 (2H, d, *J* = 8.8, H-3′/H-5′), 6.53 (1H, s, H-8), 5.14 (1H, d, *J* = 7.2, H-1″), 4.51 (1H, d, *J* = 1.3, H-1‴), 3.88 (3H, s, OCH_3_-6), 3.80 (1H, m, H-6″a), 3.62 (1H, m, H-4″), 3.52 (1H, dd, *J* = 9.5–3.4, H-3‴), 3.45 (1H, m, H-2″), 3.44 (1H, m, H-5‴), 3.42 (1H, m, H-5″), 3.36 (1H, m, H-6″b), 3.33 (1H, m, H-3″), 3.28 (1H, t, *J* = 8.0, H-4‴), 3.25 (1H, t, *J* = 9.1, H-2‴), 1.12 (3H, d, *J* = 6.2, H-6‴). ^13^C-NMR (150 MHz; CDCl_3_, *δ*(ppm)) 160.8 (C-4′), 159.6 (C-9), 153.9 (C-7), 135.1 (C-3), 132.7 (C-6), 132.4 (C-2′/C-6′), 122.7 (C-1′), 116.1 (C-3′/C-5′), 106.2 (C-10), 104.4 (C-1″), 102.4 (C-1‴), 95.2 (C-8), 78.1 (C-5″), 77.2 (C-3″), 75.7 (C-2″), 73.8 (C-4‴), 72.2 (C-3‴), 72.1 (C-4″), 71.4 (C-2‴), 69.7 (C-5‴), 68.6 (C-6″), 61.0 (-OCH_3_), 17.9 (C-6‴).

6-Methoxykaempferol (**4**), Yellow amorphous powder, ^1^H-NMR (400 MHz; DMSO-*d*_6_, *δ*(ppm), *J*(Hz)) 12.50 (1H, s, OH-5), 8.01 (2H, d, *J* = 8.9, H-2′/H-6′), 6.90 (2H, d, *J* = 8.9, H-3′/H-5′), 6.53 (1H, s, H-8), 3.73 (3H, s, OCH_3_-6). ^13^C-NMR (100 MHz; CDCl_3_, *δ*(ppm)) 176.4 (C-4), 159.4 (C-4′), 157.4 (C-7), 151.9 (C-5), 151.7 (C-9), 147.3 (C-2), 135.6 (C-3), 131.1 (C-6), 129.8 (C-2′/C-6′), 122.0 (C-1′), 115.7 (C-3′/C-5′), 103.7 (C-10), 94.0 (C-8), 60.3 (-OCH_3_). 

Luteoline-7-*O*-*β*-glucoside (**5**), Yellow amorphous powder,^1^H-NMR (400 MHz; DMSO-*d*_6_, *δ*(ppm), *J*(Hz)) 12.94 (1H, s, OH-5), 7.43 (1H, dd, *J* = 2.1–8.3, H-6′), 7.39 (1H, d, *J* = 2.1, H-2′), 6.89 (1H, d, *J* = 8.4, H-5′), 6.72 (1H, s, 3-H), 6.79 (1H, d, *J* = 2.1, H-8), 6.43 (1H, d, *J* = 2.0, H-6), 5.04 (1H, d, *J* = 7.3, H-1″), 3.71 (1H, m, H-6″a), 3.47 (1H, m, H-6″b), 3.43 (1H, m, H-5″), 3.31 (1H, t, *J* = 8.6, H-3″), 3.26 (1H, t, *J* = 7.4, H-2″), 3.17 (1H, t, *J* = 8.6, H-4″). ^13^C-NMR (100 MHz; CDCl_3_, *δ*(ppm)) 182.1 (C-4), 164.7 (C-2), 163.1 (C-7), 161.3 (C-5), 157.2 (C-9), 150.3 (C-4′), 146.0 (C-3′), 121.5 (C-1′), 119.5 (C-6′), 116.2 (C-5′), 113.6 (C-2′), 105.6 (C-10), 103.3(C-3), 101.1 (C-1″), 99.8 (C-6), 95.0 (C-8), 77.3 (C-5″), 76.5 (C-3″), 73.3 (C-2″), 69.8 (C-4″), 60.9 (C-6″). 

### 2.3. Total Phenolic and Flavonoid Contents

The total phenolic and flavonoid contents of the AcOEt and BuOH extracts were measured using the Folin–Ciocalteu and AlCl_3_ methods, respectively (Table 2).

### 2.4. Antioxidant and Photoprotective Activities

Five different in vitro methods were utilized to assess the antioxidant potential of the AcOEt and BuOH extracts of *A. ligustica*, including GOR, DPPH, CUPRAC, reducing power, and *O*-phenanthroline assays. The findings, presented as IC_50_ and A_0.50_ values measured through linear regression analysis, are given in Table 3. Furthermore, the photoprotective efficacy of both extracts was examined through the determination of their sun protection factor (SPF) within the UV-B spectrum. The recommendations of the CEC (Commission of European Communities 2006) were used to evaluate the SPF values and their classification (Table 4).

## 3. Discussion

A comprehensive examination of the LC-Q-TOF-MS findings reveals that the compounds identified in the AcOEt and BuOH extracts are categorized into five classes: flavonoids, phenolic acids, phenolic aldehydes, dicarboxylic acid, and dihydroxybenzenes. 

In this analysis, twelve flavonoids from different subclasses were identified. These included five flavonols, namely rutin, quercetin, hyperoside, rhamnetin, and fisetin; three flavones, namely apigenin, apigenin-7-*O*-glucoside, and luteolin-7-*O*-glucoside; and four flavanones, namely naringenin, naringin, hesperidin, and hesperetin. Notably, naringin and hesperidin were only detected in the BuOH extract, while naringenin was only found in the AcOEt extract. Through a comparison with the literature, it was revealed that naringin and hesperetin were previously identified in *A. abrotanoides* and *A. lingulata*, respectively [30]. Hyperoside and hesperidin were earlier detected in *A. kotschyi* and *A. lycaonica* [31], while fisetin and naringenin were found in *A. distans* [32] and *A. vermicularis* [33], respectively. These compounds have been found for the first time in *A. ligustica*. Moreover, this study is the first to our knowledge that reveals the presence of rhamnetin in the *Achillea* genus.

Additionally, eight phenolic acids, including caffeic acid, quinic acid, chlorogenic acid, protocatechuic acid, rosmarinic acid, syringic acid, *p*-coumaric acid, and 4-hydroxybenzoic acid, were identified in both extracts, with syringic acid found only in the BuOH extract. All these compounds were previously identified in the *Achillea* genus. However, the present study reports the first occurrence of syringic acid, protocatechuic acid, *p*-coumaric acid, and 4-hydroxybenzoic acid in *A. ligustica*.

Furthermore, phenolic aldehyde (vanillin) and dicarboxylic acid (malic acid), along with dihydroxybenzenes (catechol) were identified for the first time in the *A. ligustica* species. Previously, vanillin and malic acid had been found in *A. coarctata* and *A. monocephala* [34], while catechol was identified in *A. millefolium* [35].

From the AcOEt and BuOH extracts obtained from *A. ligustica* aerial parts, five compounds were isolated. NMR spectra analysis disclosed the presence of flavone structures, identifying compound **1** as apigenin and compound **2** as luteolin. Notably, these compounds were previously isolated from *A. ligustica* [1,10,17]. Additionally, three other flavonoids were isolated from the BuOH extract, which were identified as 6-methoxykaempferol-3-*O*-rutinoside **3**, 6-methoxykaempferol **4**, and luteoline-7-*O*-*β*-glucoside **5**. The two compounds **3** and **4** are identified for the first time from the *Achillea* genus, whereas compound **5** is a common compound in *A. ligustica* [10].

The TPC and TFC analysis revealed that both extracts exhibited significant levels of phenolic and flavonoid compounds with 465.47 ± 1.02 μg GAE/mg and 225.63 ± 0.59 μg EQ/mg for the AcOEt extract and 258.12 ± 1.02 μg GAE/mg and 147.50 ± 2.21 μg EQ/mg for the BuOH extract, respectively. In a previous study conducted on the same plant, the measured TPC of the hydroalcoholic extract was lower than our result [2]. Furthermore, our findings were considerably higher than those reported for the species *A. fragrantissima* [36].

To provide a comprehensive knowledge of the antioxidant activity of the AcOEt and BuOH extracts, five different methods were employed. The results from all assays indicated that both extracts demonstrated potent antioxidant activity, with the AcOEt extract showing greater activity than the BuOH extract (Table 3). Additionally, the AcOEt extract exhibited superior activity compared to the standard BHA, BHT, and α-tocopherol in the GOR, CUPRAC, and reducing power assays, respectively. These results affirm the correlation between the phenolic and flavonoid contents and antioxidant activities [37]. The AcOEt and BuOH extracts revealed strong antioxidant capacities when compared to the hydroalcoholic extract of the same plant, as evaluated by the DPPH and reducing power assays [2]. Meanwhile, the antioxidant potential measured by the phenanthroline method in the same study yielded results similar to our findings.

Skin damage caused by UV radiation ranks among the most prevalent concerns throughout the world. Research has demonstrated that photoprotective agents, particularly sunscreens, are vital in decreasing the occurrence of skin disorders, such as pigmentation issues and premature aging caused by UV exposure [38]. Numerous recent studies have explored natural substances as potential resources for sunscreen due to their capacity to absorb UV radiation and their antioxidant properties [24]. In the present study, both examined extracts demonstrated a potent ability to absorb UV light (Table 4), with values higher than those of CSS 1 but lower than those of CSS 2, the standards used for this test. The significant UV absorption of both extracts is due to their richness in phenolic and flavonoid compounds. This conclusion is supported by research which recognized that phenolic compounds are potent sun filters, offering substantial photoprotective benefits [39]. As a consequence, the AcOEt and BuOH extracts of *A. ligustica* can be utilized as sun protection agents in sunscreen products within the cosmetic industry or pharmaceutical formulations. The photoprotective properties of *A. ligustica* have not been studied before. Therefore, the findings of this research provide a novel contribution to existing research.

## 4. Material and Methods

### 4.1. Plant Material

*A. ligustica* aerial parts were harvested during the flowering period (May 2018) at Mila in Northeast Algeria (36°34′ N, 5°57′ E). The plant was authenticated by Pr. Hocine Laouar (University of Sétif 1, Sétif, Algeria). The collected species was dried at room temperature far from light. A voucher specimen was kept in the PHYSYNOR laboratory (Chemistry department, University of Mentouri Constantine 1) under N° AL012.

### 4.2. Extraction and Isolation

The powdered aerial parts (500 g) of *A. ligustica* were defatted with cyclohexane solvent for 48 h. After filtration, the residue was macerated with Ethanol–H_2_O (7:3) solution for 48 h three times. The obtained extracts were combined and concentrated under reduced pressure until dry to produce a hydroalcoholic crude extract, which was then dissolved in distilled water. After filtration, the resulting aqueous phase was extracted sequentially with chloroform, ethyl acetate, and 1-butanol. These organic layers were evaporated until dry, yielding the corresponding extracts: 6.03 g of CHCl_3_, 3.99 g of AcOEt, and 11.68 g of BuOH.

### 4.3. HPLC-Q-TOF-MS Analysis

The MS analyses of AcOEt and BuOH extracts were performed by a 1290 infinity II liquid chromatography system (Agilent Technologies) equipped with a binary pump (model G7120A), an autosampler, a source ionization by electrospray (ESI, Dual Agilent Jet Stream model), a diode array detector (DAD), and a quadrupole time-of-flight mass spectrometry analyzer (Q-TOF, model 6546). A column (2.1 × 100 mm, 1.8 µm) (Agilent Technologies, Palo Alto, CA, USA) was used. Water with 0.1% formic acid (solvent A) and acetonitrile (solvent B) were used as gradient elution. The flow rate was 0.5 mL/min, the column temperature was maintained at 25 °C, and the injection volume was 4 μL. The solvent gradient involved in the B mobile phase was as follows: 0–1 min, 2% B; 1–30 min, 95% B; and 30–41 min, 2% B. The separated compounds were sequentially analyzed, initially using a DAD and subsequently using a mass spectrometry detector. Mass spectra were acquired over a mass range from *m*/*z* 100 to 2500, utilizing a negative ionization mode. The structural identification of the compounds was determined by comparing their retention times and mass spectra with standard compounds [39].

### 4.4. Isolation and Purification of Compounds

To further explore the constituents of *A. ligustica*, the two extracts were separated using chromatographic techniques. Consequently, 3.5 g of the AcOEt extract was fractioned by silica gel column chromatography (type 60, 0.063–0.200 mm, Merck, Darmstadt, Germany) using cyclohexane-ethyl acetate-methanol as system solvents. Similar fractions were combined after TLC analysis over silica gel GF_254_ plates to give 10 fractions (F_1_ to F_10_). Two compounds, **1** (2.8 mg) and **2** (6.6 mg), were isolated from fractions F_4_ (89.4 mg) and F_6_ (69.9 mg), respectively, after the treatment of the yellow precipitate with chloroform and subsequently with acetone.

On the other hand, a 6 g portion of the BuOH extract was subjected to polyamide SC6 column chromatography eluting with a gradient of H_2_O–MeOH (0% to 100%). After combining similar fractions based on TLC analysis over cellulose (CEL 400 plates, Merck) visualized under UV light at 365 nm, 15 fractions were obtained. Fraction F_4_ (283.7 mg) was subjected to chromatographic separation using the Sephadex LH-20 column with methanol as the eluent, yielding five subfractions. Subsequently, the subfraction F_4_(2) was separated by preparative silica gel GF_254_ TLC using AcOEt-MeOH-H_2_O (6:1:1) as an elution system to obtain compound **3** (6.1 mg), which was purified over a Sephadex LH20 column with MeOH as the eluent. Additionally, Fraction F_6_ (155.1 mg) was also purified using a Sephadex LH-20 column with MeOH as the eluent, resulting in the isolation of compound **4** (3.8 mg). Meanwhile, compound **5** (5.8 mg) was isolated as a precipitate from fraction F_8_ (190.7 mg), further purified by precipitation in methanol.

### 4.5. Determination of Total Phenolics and Flavonoids Contents

#### 4.5.1. Total Phenolic Content (TPC)

The TPC was estimated spectrophotometrically using the Folin–Ciocalteu (FCR) reagent [40,41]. A concentration of 1 mg/mL of the AcOEt and BuOH extracts was used in the analysis. A total of 20 μL of each extract solution was mixed with 100 μL of FCR (1:10) and 75 μL of sodium carbonate (7.5%). The resulting solution was left in the dark at room temperature for 2 h. After incubation, the absorbance was measured against a blank at 765 nm. The total polyphenol content was calculated from the calibration curve of a gallic acid standard solution, and the results were expressed as micrograms of gallic acid equivalents per milligrams of extract (μg GAE/mg).

#### 4.5.2. Total Flavonoid Content (TFC)

The TFC was assessed spectrophotometrically according to the method described by Topçu et al. [42]. Each extract solution (50 μL) was diluted with 130 μL of MeOH and then mixed with 10 μL of potassium acetate (1M) and 10 μL of aluminum nitrate solution (10%). The resulting solution was left in the dark at room temperature, and after 40 min of incubation, the absorbance was measured against a blank at 415 nm. The total flavonoid content was calculated from the calibration curve of a quercetin standard solution. The results were expressed as micrograms of quercetin equivalents per milligrams of extract (μg QE/mg).

### 4.6. Estimation of Antioxidant Activities 

#### 4.6.1. DPPH Free Radical Scavenging Activity

The DPPH scavenging ability of the AcOEt and BuOH extracts was performed spectrophotometrically [43]. To 40 μL of sample solutions (AcOEt, BuOH extracts, and the standard BHA) at different concentrations, 160 μL of DPPH solution (0.1 mM) in methanol was added, and the mixture was left in the dark at room temperature. After 30 min of incubation, the absorbance of the mixture was measured at 517 nm. The results were provided as IC_50_ (μg/mL) values. The inhibition percentage of DPPH radical was calculated using the following formula:

$$\%\mathrm{inhibition}=\frac{A_{\mathrm{control}}-A_{\mathrm{sample}}}{A_{\mathrm{control}}}\times 100$$

A_control_: Absorbance of control (contained in all reagents except the test extract or standard). 

A_sample_: Absorbance of the test extract or standard. 

#### 4.6.2. Galvinoxyl (GOR) Scavenging Activity

The galvinoxyl free radical (GOR) antioxidant test of the AcOEt and BuOH extracts was evaluated spectrophotometrically using the Shi et al. [44] method. A volume of 40 μL of sample solutions (AcOEt, BuOH extracts, and the standards BHA, BHT) at various concentrations was mixed with 160 μL of galvinoxyl methanolic solution (0.1 mM). After incubation in the dark at room temperature for 2 h, the absorbance was measured at 428 nm. The results were expressed as IC_50_ (μg/mL) values, and the above formula was used to calculate the inhibition percentage of GOR radicals.

#### 4.6.3. Cupric Reducing Antioxidant Capacity (CUPRAC) Activity

The CUPRAC activity of the AcOEt and BuOH extracts was assessed spectrophotometrically using the method described by Apak et al. [45]. Briefly, 60 μL of CH_3_COONH_4_ buffer (1 M, pH 7), 50 μL of neocuproine solution (7.5 mM) in methanol, and 50 μL of CuCl_2_ (10 mM) were added to 40 μL of sample solutions (extract and standard) at different concentrations. After incubation at room temperature for 60 min, the absorbance was measured at 450 nm using the microplate reader. BHA and BHT were used as standards, and the results were given as A_0.50_ value.

#### 4.6.4. Reducing Power Activity

The reducing power activity was investigated spectrophotometrically according to the procedure outlined by Oyaizu [46]. For this assay, a volume of 10 μL of sample solutions (AcOEt, BuOH extracts, and standards) at different concentrations was mixed with 40 μL of phosphate buffer solution (0.2 M, pH 6.6) and 50 μL of potassium ferricyanide (1%). Later, after incubation for 20 min at 50 °C, 50 μL of trichloroacetic acid (10%), 40 μL of distilled water, and 10 μL of FeCl_3_ solution (0.1%) were added. The absorbance was then measured at 700 nm using a microplate reader. Ascorbic acid, tannic acid, and α-tocopherol were used as standards, and the results were expressed as A_0.50_ values.

#### 4.6.5. *O*-Phenanthroline Activity

The phenanthroline chelating activity of AcOEt and BuOH extracts was performed spectrophotometrically according to Szydlowska-Czerniak et al. [47]. The procedure consisted of adding 50 μL of FeCl_3_ solution (0.2%), 30 μL of *O*-phenanthroline (0.5%), and 110 μL of methanol to 10 μL of sample solutions (extracts and standards) at different concentrations. The microplate was incubated for 20 min at 30 °C, and the absorbance was read at 510 nm. BHA and BHT were used as standards, and the results were provided as A_0.50_ value.

### 4.7. Photoprotective Activity

The in vitro photoprotective activity of the AcOEt and BuOH extracts against UV damage was evaluated according to the method established by Mansur et al. [48], with results expressed as the sun protection factor (SPF). In this assay, 2 mg of each extract was dissolved in 1 mL of methanol. Afterward, a volume of 200 μL of sample solutions was placed into a microplate. The absorbance was measured in the range 290–320 nm (UV-B wavelength), with increments of 5 nm. The SPF value was calculated using the following formula:

$$FPS=CF\times \sum_{290}^{320}EE\left(\lambda \right)\times I\left(\lambda \right)\times Abs\left(\lambda \right)$$

CF: correction factor (=10); EE(λ): erythemal effect spectrum; I(λ): solar intensity spectrum; Abs(λ): absorbance of sunscreen product. The values of EE(λ) × I(λ) are constants determined by Sayre et al. [49].

### 4.8. Statistical Data Analysis

All results are expressed as mean values ± standard deviation (SD) of three measurements. The IC_50_ (inhibition concentration at 50%) and A_0.50_ values (the concentration indicating 0.50 absorption) were calculated by the linear regression analysis from the curve [% Inhibition = f(concentration)] for IC_50_ and [Absorbance = f(concentration)] for A_0.50_. Differences between means were assessed using Student’s *t*-test, and *p*-values < 0.05 were found to be statistically significant.

## 5. Conclusions

This study delved into the metabolite composition of the AcOEt and BuOH extracts of *A. ligustica* aerial parts harvested from Algeria. The phytochemical examination using HPLC-Q-TOF-MS reveals a diverse array of bioactive compounds, such as flavonoids, phenolic acids, phenolic aldehydes, dicarboxylic acids, and dihydroxybenzenes. Furthermore, in addition to three flavones previously identified in this species, two flavonols known as 6-methoxykaempferol-3-*O*-rutinoside (**3**) and 6-methoxykaempferol (**4**) were separated for the first time from the *Achillea* genus. The study also highlighted the significant antioxidant and photoprotective properties of the plant, underscoring its potential as a promising natural resource for the development of pharmaceutical and cosmetic products.

## Figures and Tables

**Table 1:** HPLC-MS data of compounds identified in AcOEt and BuOH extracts of *A. ligustica*.

No.	Compounds	Rt (min)	Molecular Formula	*m*/*z* (Molecular Ion)
1	Quinic acid	3.22	C_7_H_12_O_6_	192.0634
2	Malic acid	3.31	C_4_H_6_O_5_	134.0216
3	Protocatechuic acid	7.74	C_7_H_6_O_4_	154.0267
4	Syringic acid ^a^	7.97	C_9_H_10_O_5_	198.0530
5	Catechol	8.72	C_6_H_6_O_2_	110.0366
6	4-Hydroxybenzoic acid	10.05	C_7_H_6_O_3_	138.0318
7	Chlorogenic acid	11.63	C_16_H_18_O_9_	354.0951
8	Caffeic acid	12.60	C_9_H_8_O_4_	180.0424
9	*p*-Coumaric acid	14.73	C_9_H_8_O_3_	164.0469
10	Naringin ^a^	16.44	C_27_H_32_O_14_	580.1786
11	Hesperetin	16.84	C_16_H_14_O_6_	302.0793
12	Rutin	17.36	C_27_H_30_O_16_	610.1535
13	Hyperoside	17.41	C_21_H_20_O_12_	464.0956
14	Hesperidin ^a^	17.49	C_28_H_34_O_15_	610.1875
15	Vanillin	17.97	C_8_H_8_O_3_	152.0475
16	Apigenin-7-*O*-glucoside	18.21	C_21_H_20_O_10_	432.1056
17	Luteolin-7-*O*-glucoside	18.73	C_21_H_20_O_11_	448.1004
18	Naringenin ^b^	20.42	C_15_H_12_O_5_	272.0683
19	Quercetin	20.47	C_15_H_10_O_7_	302.0424
20	Fisetin	21.16	C_15_H_10_O_6_	286.0475
21	Rhamnetin	21.43	C_16_H_12_O_7_	316.0583
22	Apigenin	22.63	C_15_H_10_O_5_	270.0529
23	Rosmarinic acid	26.31	C_18_H_16_O_8_	360.0836

Rt: Retention time; ^a^: not detected in ethyl acetate extract; ^b^: not detected in 1-butanol extract.

**Table 2:** TPC and TFC of AcOEt and BuOH extracts of *A. ligustica*.

Extract	TPC (μg GAE/mg)	TFC (μg EQ/mg)
AcOEt	465.47 ± 1.02	225.63 ± 0.59
BuOH	258.12 ± 1.02	147.50 ± 2.21

**Table 3:** Antioxidant activity of AcOEt and BuOH extracts of *A. ligustica*.

Samples	IC_50_ (μg/mL)		A_0.50_ (μg/mL)		
	DPPH	GOR	CUPRAC	Reducing Power	Phenanthroline
AcOEt	7.13 ± 0.15	4.57 ± 0.10	5.61 ± 0.06	13.14 ± 2.20	5.17 ± 0.27
BuOH	13.06 ± 0.60	8.33 ± 0.30	9.56 ± 0.28	29.12 ± 1.16	8.83 ± 0.50
BHA	5.73 ± 0.41	5.38 ± 0.06	3.64 ± 0.19	NT	0.93 ± 0.07
BHT	NT	3.32 ± 0.18	9.62 ± 0.87	NT	2.24 ± 0.17
Ascorbic acid	NT	NT	NT	6.77 ± 1.15	NT
Tannic acid	NT	NT	NT	5.39 ± 0.91	NT
α-Tocopherol	NT	NT	NT	34.93 ± 2.38	NT

BHA: Butylated hydroxyanisole; BHT: Butylated hydroxytoluene; NT: Not tested.

**Table 4:** Photoprotective activity of AcOEt and BuOH extracts of *A. ligustica*.

Extract	SPF	Protection Category *
AcOEt	48.08 ± 0.01	High protection
BuOH	48.08 ± 0.05	High protection
CSS 1	44.22 ± 0.35	High protection
CSS 2	50.11 ± 0.53	High protection

* Classification following the Commission of European Communities 2006 recommendation. CSS 1: Commercial sunscreen 1; CSS 2: Commercial sunscreen 2.

## Data Availability

The data from this study can be found in the main text and the Supplementary Materials.

## Supplementary Materials

The following supporting information can be downloaded at https://www.mdpi.com/article/10.3390/molecules29174112/s1, Figure S1: ^1^H-NMR spectrum of compound **1** (DMSO-*d*_6_, 400 MHz); Figure S2: ^13^C-NMR spectrum of compound **1** (DMSO-*d*_6_, 400 MHz); Figure S3: HSQC spectrum of compound **1** (DMSO-*d*_6_, 400 MHz); Figure S4: HMBC spectrum of compound **1** (DMSO-*d*_6_, 400 MHz); Figure S5: ^1^H-NMR spectrum of compound **2** (DMSO-*d*_6_, 400 MHz); Figure S6: ^1^H-NMR spectrum of compound **2** (DMSO-*d*_6_, 400 MHz); Figure S7: ^13^C-NMR spectrum of compound **2** (DMSO-*d*_6_, 400 MHz); Figure S8: ^1^H-NMR spectrum of compound **3** (CD_3_OD, 600 MHz); Figure S9: ^1^H-NMR spectrum of compound **3** (CD_3_OD, 600 MHz); Figure S10: ^13^C-NMR spectrum of compound **3** (CD_3_OD, 600 MHz); Figure S11: HSQC spectrum of compound **3** (CD_3_OD, 600 MHz); Figure S12: HMBC spectrum of compound **3** (CD_3_OD, 600 MHz); Figure S13: ^1^H-NMR spectrum of compound **4** (DMSO-*d*_6_, 400 MHz); Figure S14: ^13^C-NMR spectrum of compound **4** (DMSO-*d*_6_, 400 MHz); Figure S15: HSQC spectrum of compound **4** (DMSO-*d*_6_, 400 MHz); Figure S16: HMBC spectrum of compound **4** (DMSO-*d*_6_, 400 MHz); Figure S17: ^1^H-NMR spectrum of compound **5** (DMSO-*d*_6_, 400 MHz); Figure S18: ^1^H-NMR spectrum of compound **5** (DMSO-*d*_6_, 400 MHz); Figure S19: ^13^C-NMR spectrum of compound **5** (DMSO-*d*_6_, 400 MHz); Figure S20: HSQC spectrum of compound **5** (DMSO-*d*_6_, 400 MHz); Figure S21: HMBC spectrum of compound **5** (DMSO-*d*_6_, 400 MHz); Figure S22: COSY spectrum of compound **5** (DMSO-*d*_6_, 400 MHz); Figure S23: Base peak chromatogram of the AcOEt extract by HPLC-Q-TOF-MS in the negative ionization mode; Figure S24: Base peak chromatogram of the BuOH extract by HPLC-Q-TOF-MS in the negative ionization mode.
