# Supplementary material for: Phenolic Constituents, Photoprotective Effect, and Antioxidant Capacities of Achillea ligustica All
Source: Molecules. 2024 Aug 30;29(17):4112. doi: 10.3390/molecules29174112 (PMC11397229; doi:10.3390/molecules29174112)
Supplement: Supplementary file 1 [file molecules-29-04112-s001.zip › molecules-3157985-supplementary.pdf]

Phenolic Constituents, Photoprotective Effect, and Antioxidant Capacities of *Achillea ligustica* All.

Azza Bouteche [1], Ahmed Touil [1], Salah Akkal [2], Chawki Bensouici [3] and Gema Nieto [4,*]

[1] Laboratory of Natural Product from Plants and Organic Synthesis, Department of Chemistry, University of Mentouri Constantine 1, Constantine 25000, Algeria
[2] Valorization of Natural Resources, Bioactive Molecules and Biological Analysis Unit, Department of Chemistry, University of Mentouri Constantine 1, Constantine 25000, Algeria
[3] Centre de Recherche en Biotechnologie Constantine, Constantine 25000, Algeria
[4] Department of Food Technology, Nutrition and Food Science, Veterinary Faculty, University of Murcia, Regional Campus of International Excellence "Campus Mare Nostrum", Campus de Espinardo, 30100 Murcia, Spain
* Correspondence: gnieto@um.es

Supplementary materials

Figure S1. ^{1}H-NMR spectrum of compound **1** (DMSO-d_6, 400 MHz)……..02
Figure S2. ^{13}C-NMR spectrum of compound **1** (DMSO-d_6, 400 MHz)……02
Figure S3. HSQC spectrum of compound **1** (DMSO-d_6, 400 MHz)……..03
Figure S4. HMBC spectrum of compound **1** (DMSO-d_6, 400 MHz)……..03
Figure S5. ^{1}H-NMR spectrum of compound **2** (DMSO-d_6, 400 MHz)……04
Figure S6. ^{1}H-NMR spectrum of compound **2** (DMSO-d_6, 400 MHz)……..04
Figure S7. ^{13}C-NMR spectrum of compound **2** (DMSO-d_6, 400 MHz)……05
Figure S8. ^{1}H-NMR spectrum of compound **3** (CD_3OD, 600 MHz)……05
Figure S9. ^{1}H-NMR spectrum of compound **3** (CD_3OD, 600 MHz)……06
Figure S10. ^{13}C-NMR spectrum of compound **3** (CD_3OD, 600 MHz)……..06
Figure S11. HSQC spectrum of compound **3** (CD_3OD, 600 MHz)……...07
Figure S12. HMBC spectrum of compound **3** (CD_3OD, 600 MHz)……..07
Figure S13. ^{1}H-NMR spectrum of compound **4** (DMSO-d_6, 400 MHz)……08
Figure S14. ^{13}C-NMR spectrum of compound **4** (DMSO-d_6, 400 MHz)……08
Figure S15. HSQC spectrum of compound **4** (DMSO-d_6, 400 MHz)……09
Figure S16. HMBC spectrum of compound **4** (DMSO-d_6, 400 MHz)……...09
Figure S17. ^{1}H-NMR spectrum of compound **5** (DMSO-d_6, 400 MHz)……10
Figure S18. ^{1}H-NMR spectrum of compound **5** (DMSO-d_6, 400 MHz)……10
Figure S19. ^{13}C-NMR spectrum of compound **5** (DMSO-d_6, 400 MHz)……...11
Figure S20. HSQC spectrum of compound **5** (DMSO-d_6, 400 MHz)……11
Figure S21. HMBC spectrum of compound **5** (DMSO-d_6, 400 MHz)……...12
Figure S22. COSY spectrum of compound **5** (DMSO-d_6, 400 MHz)……12
Figure S23. Base peak chromatogram of the AcOEt extract by HPLC-Q-TOF-MS in the negative ionization mode……....13
Figure S24. Base peak chromatogram of the BuOH extract by HPLC-Q-TOF-MS in the negative ionization mode……13

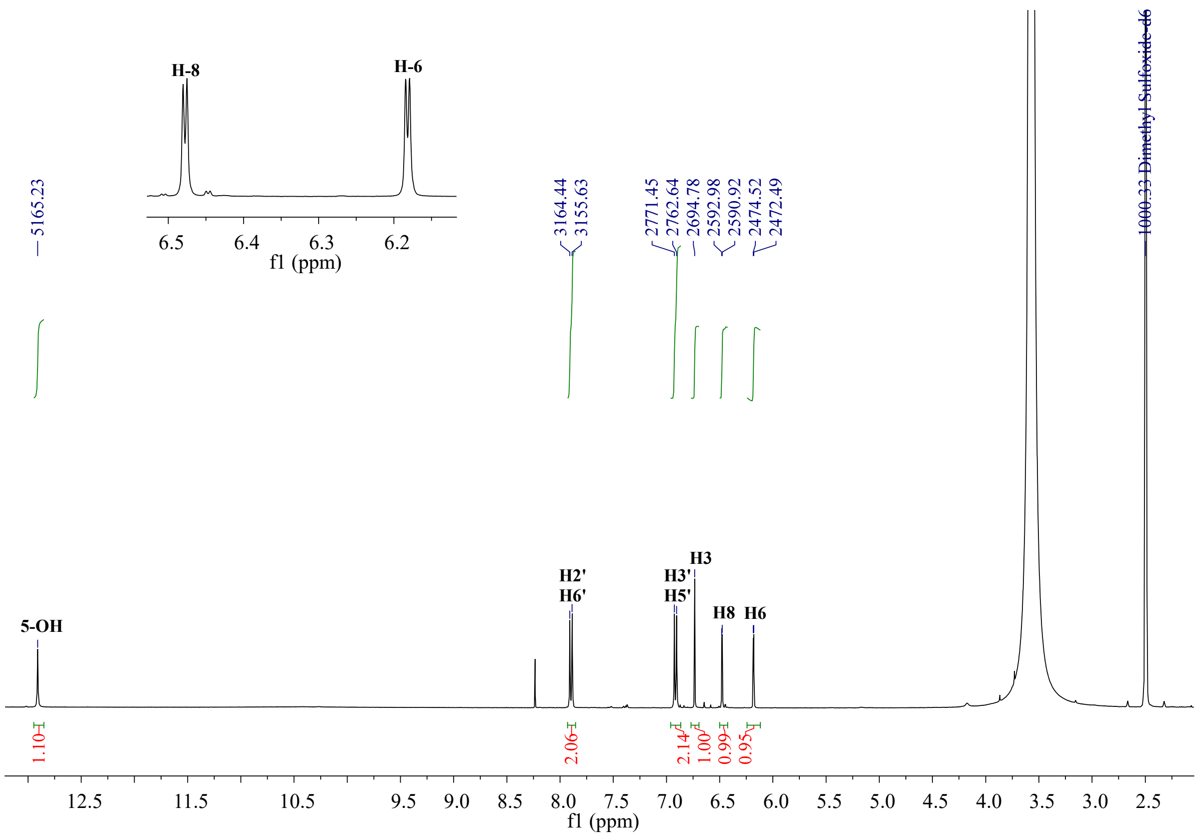

Figure S1. ^{1}H-NMR spectrum of compound **1** (DMSO-d_6, 400 MHz)

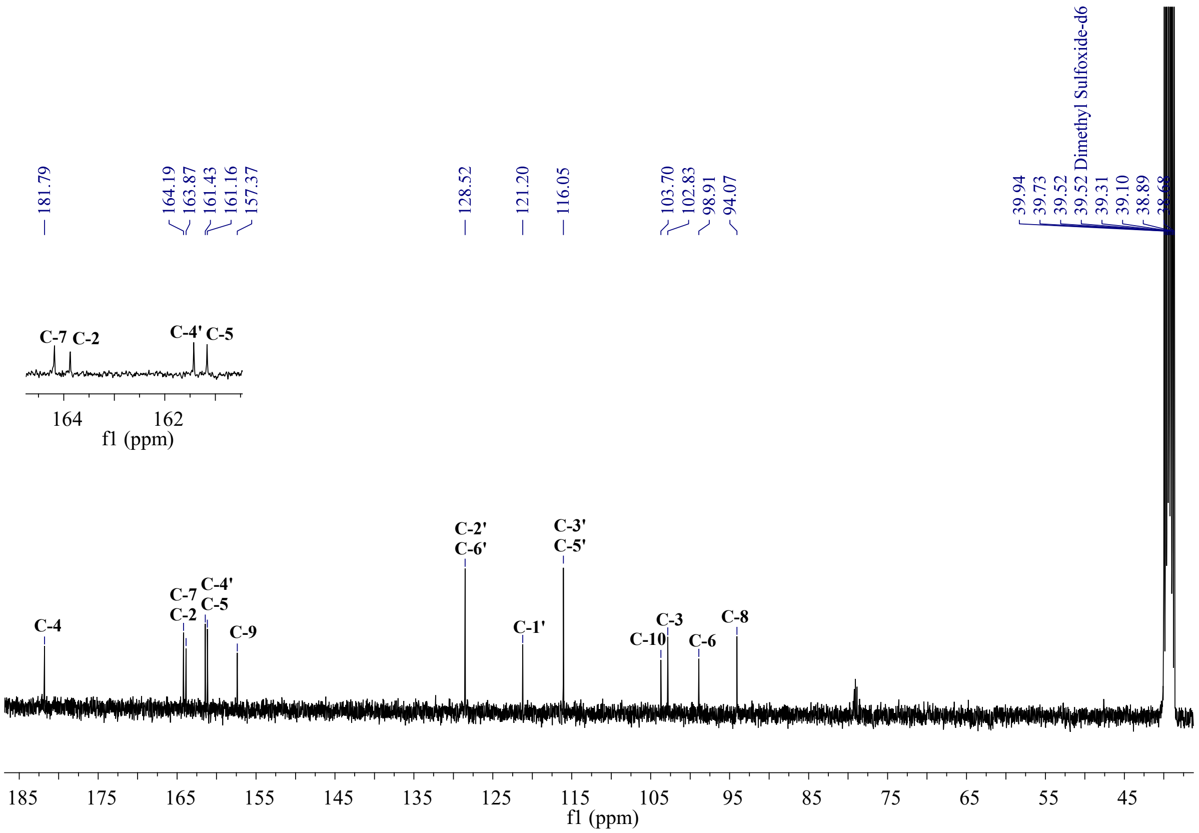

Figure S2. ^{13}C-NMR spectrum of compound **1** (DMSO-d_6, 400 MHz)

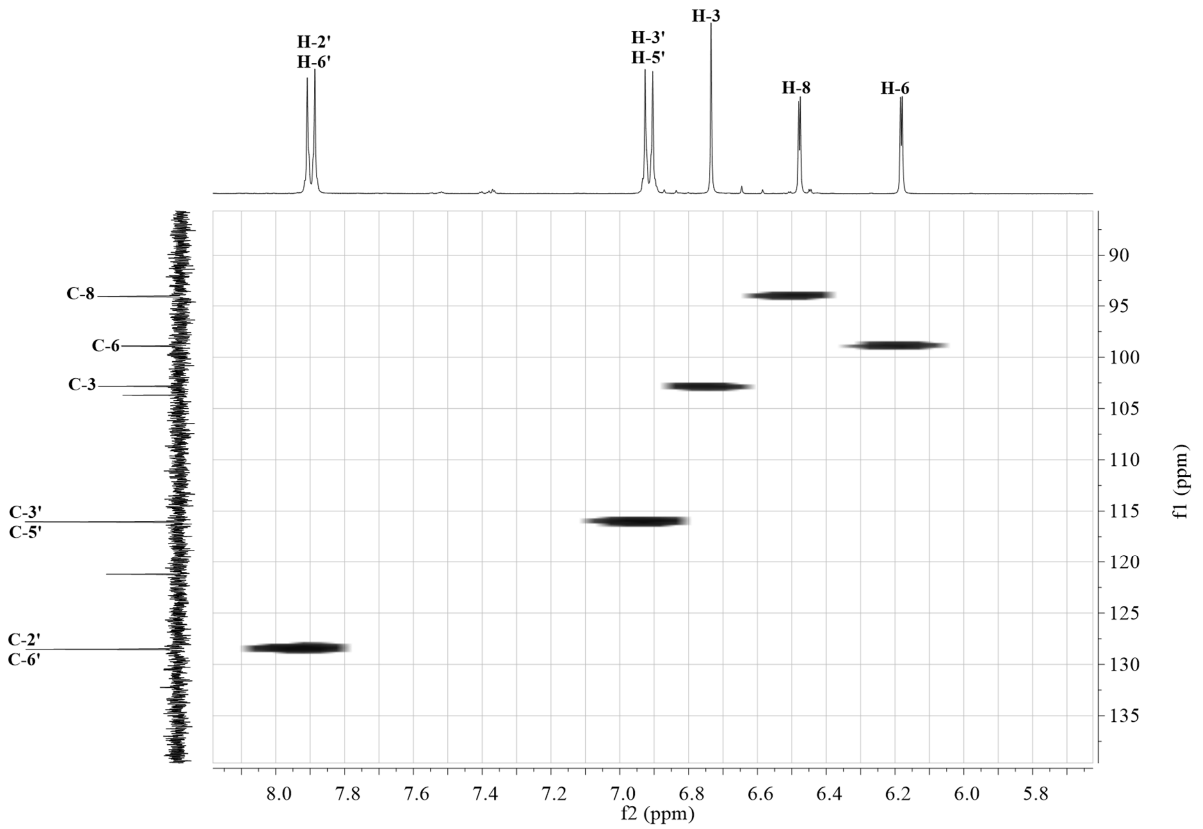

Figure S3. HSQC spectrum of compound **1** (DMSO-d_6, 400 MHz)

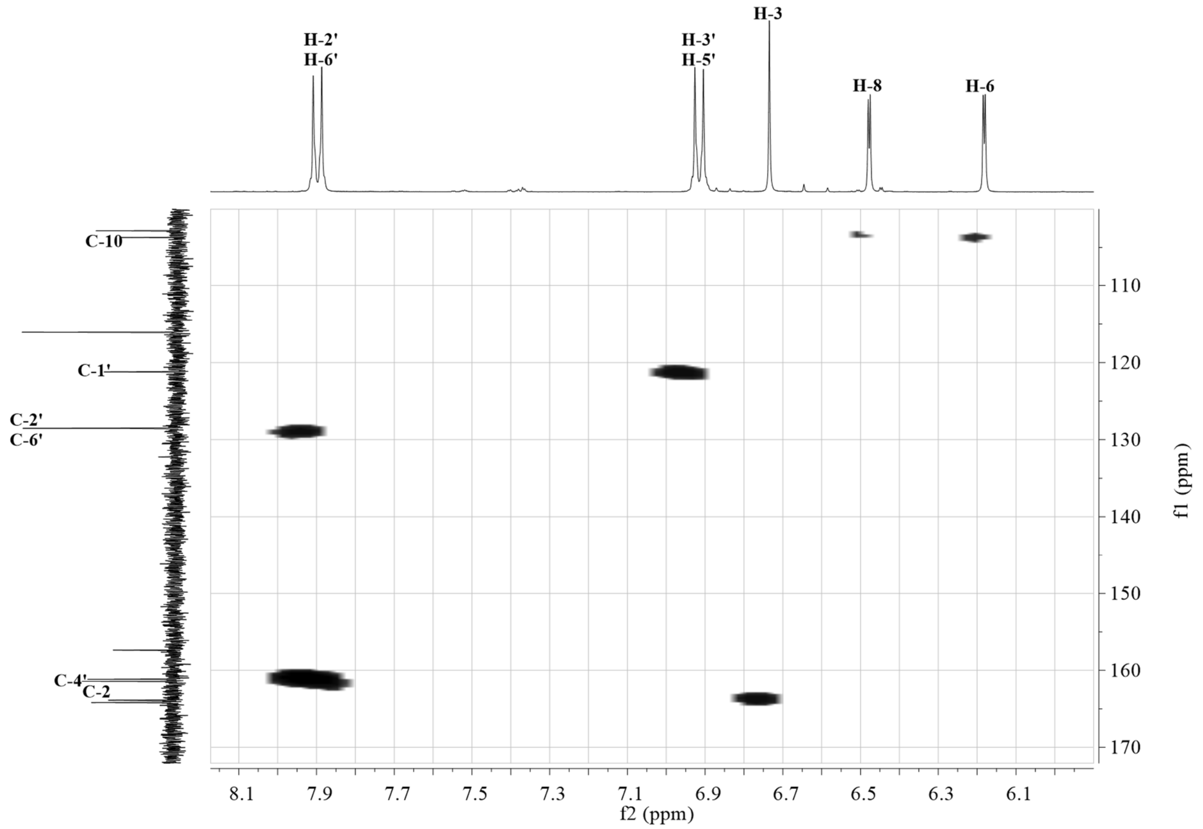

Figure S4. HMBC spectrum of compound **1** (DMSO-d_6, 400 MHz)

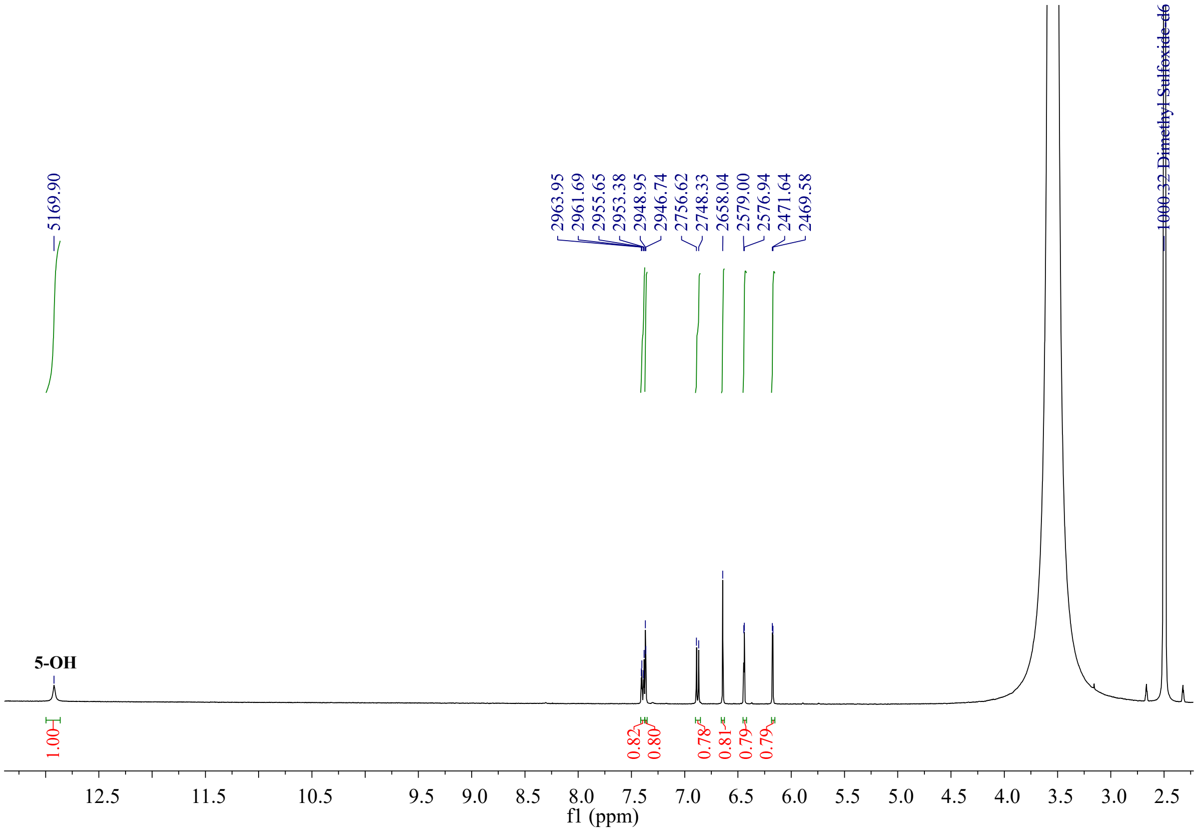

Figure S5. ^{1}H-NMR spectrum of compound **2** (DMSO-d_6, 400 MHz)

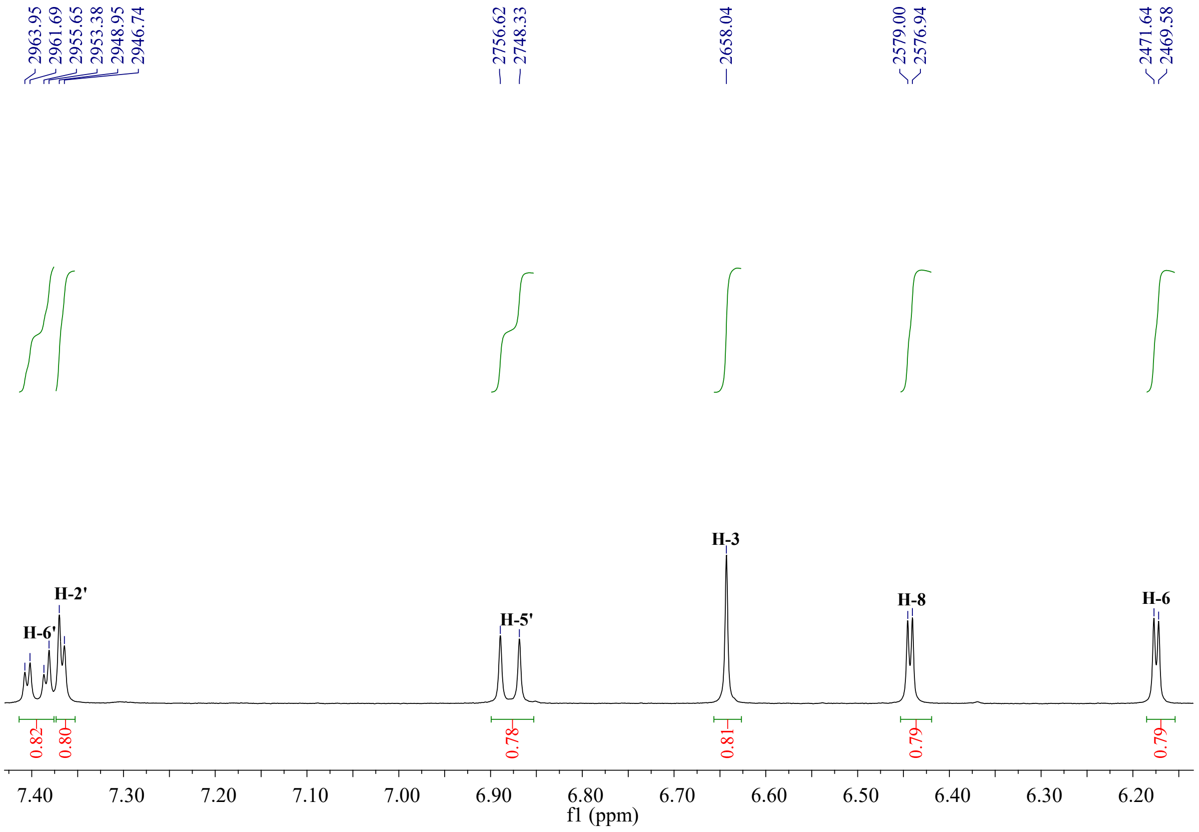

Figure S6. ^{1}H-NMR spectrum of compound **2** (DMSO-d_6, 400 MHz)

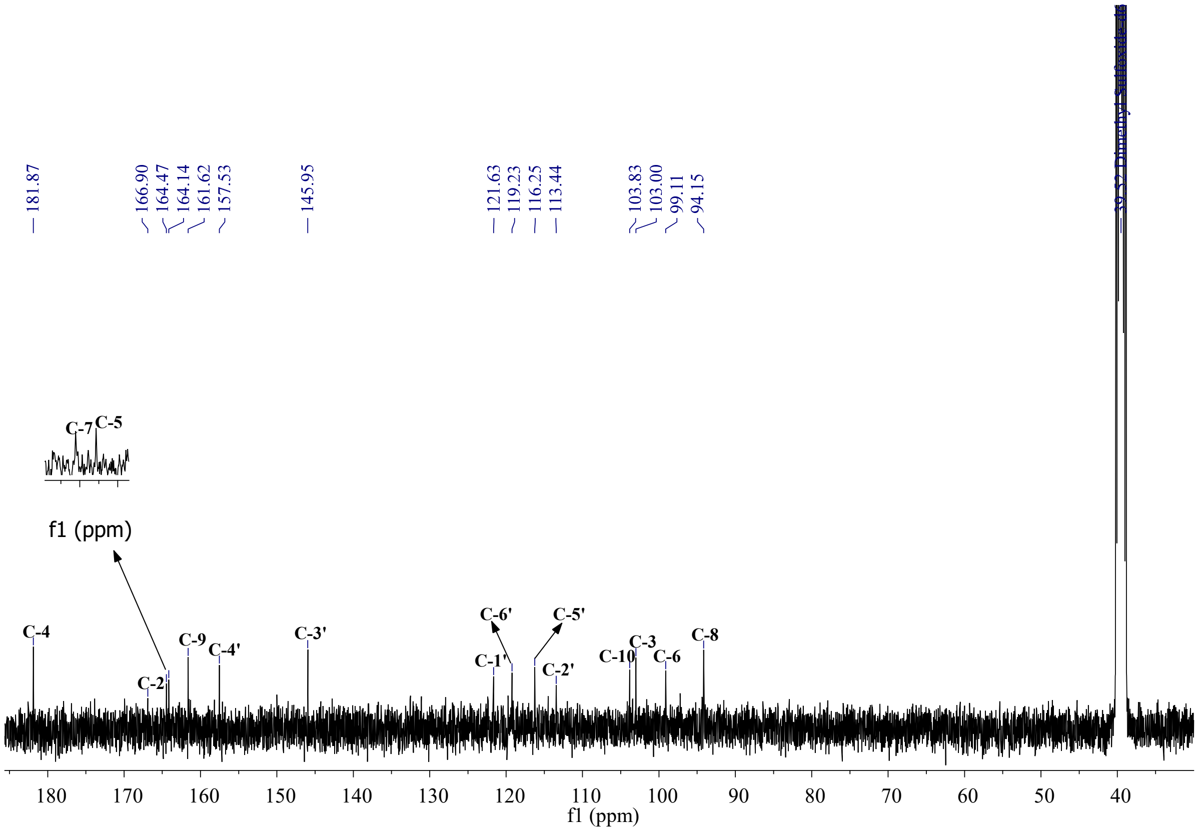

Figure S7. ^{13}C-NMR spectrum of compound **2** (DMSO-d_6, 400 MHz)

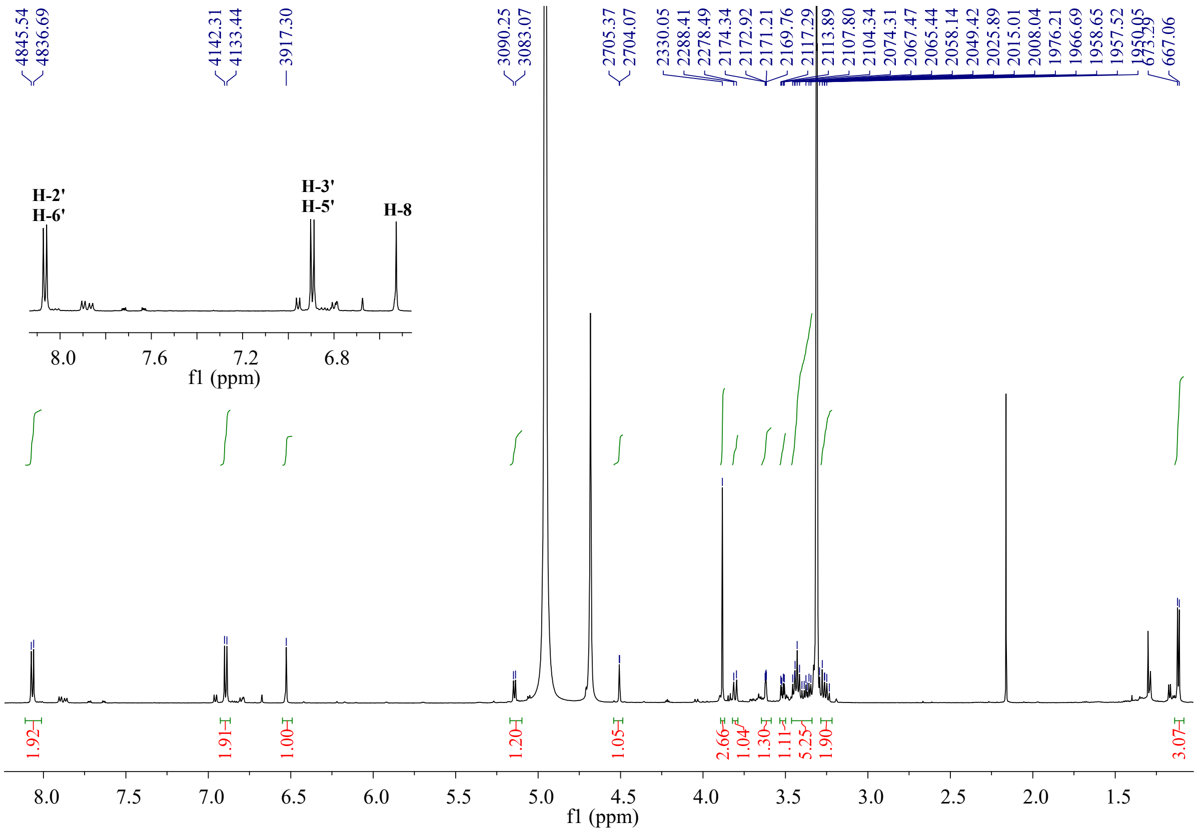

Figure S8. ^{1}H-NMR spectrum of compound **3** (CD_3OD, 600 MHz)

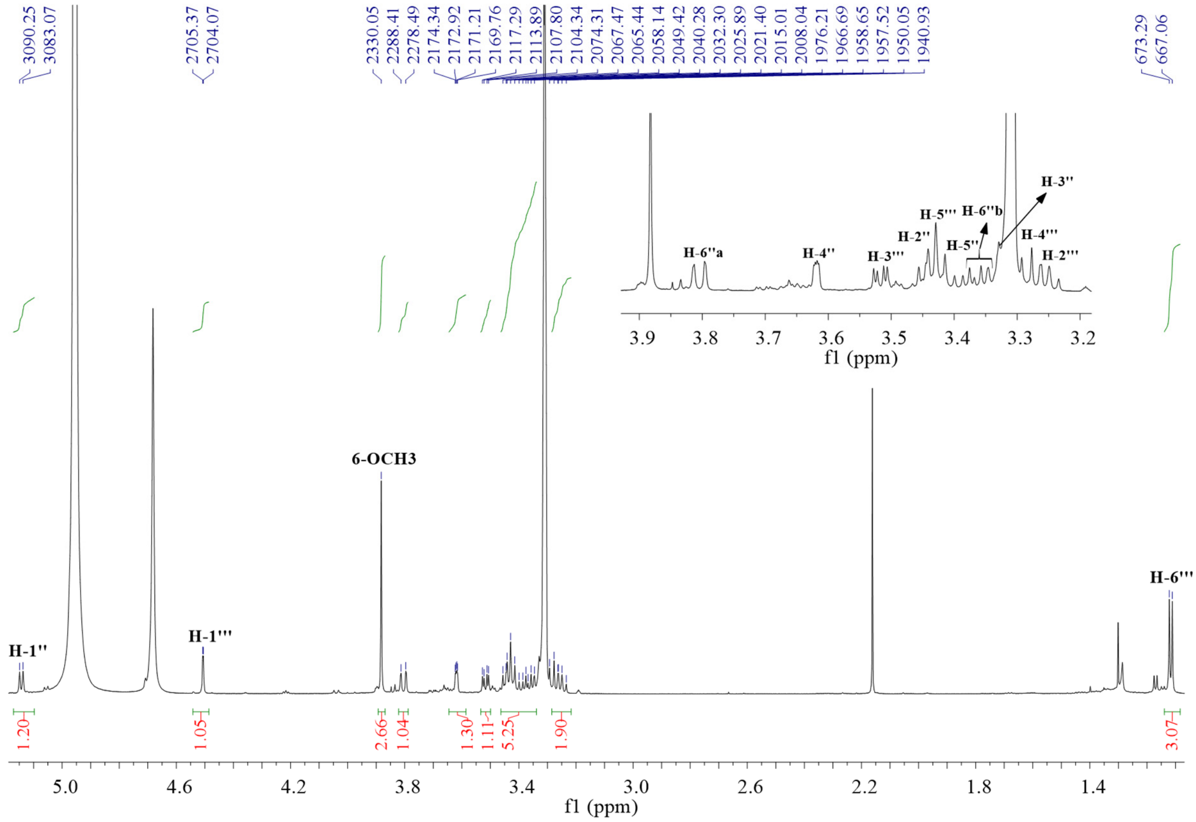

Figure S9. ^{1}H-NMR spectrum of compound **3** (CD_3OD, 600 MHz)

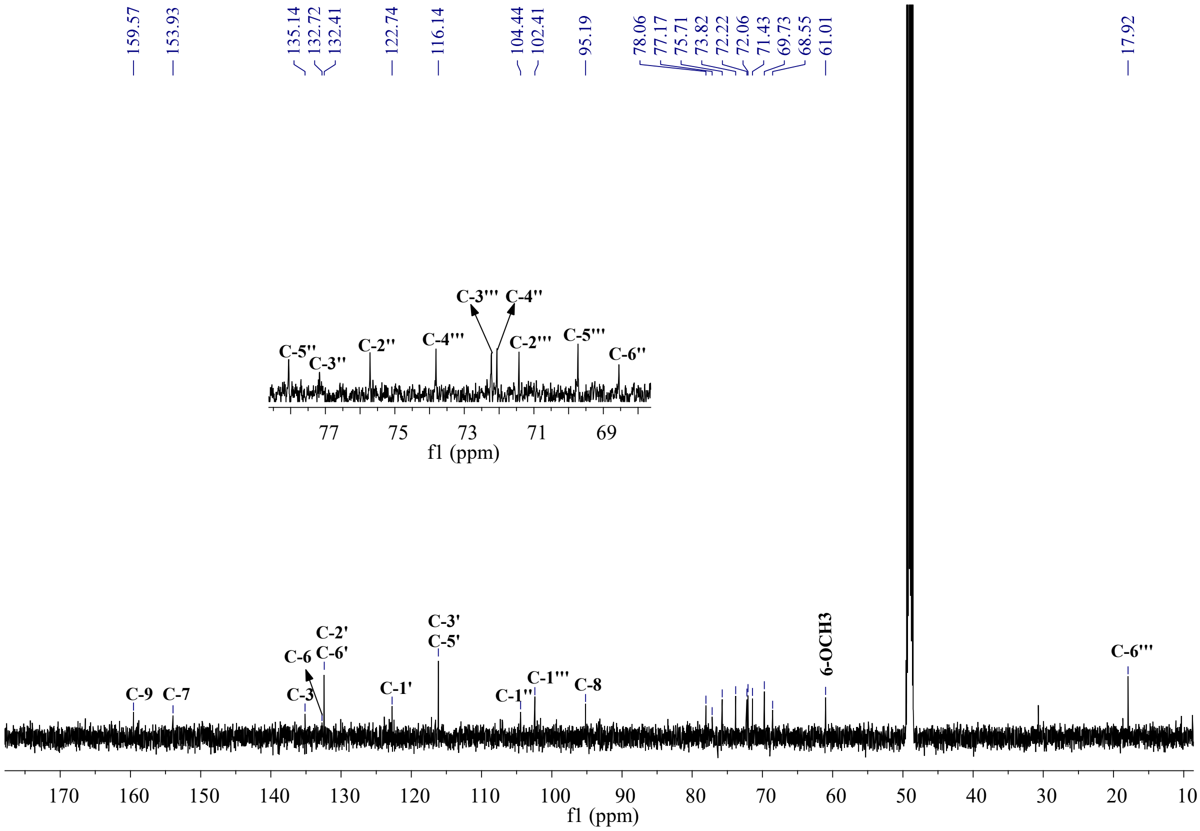

Figure S10. ^{13}C-NMR spectrum of compound **3** (CD_3OD, 600 MHz)

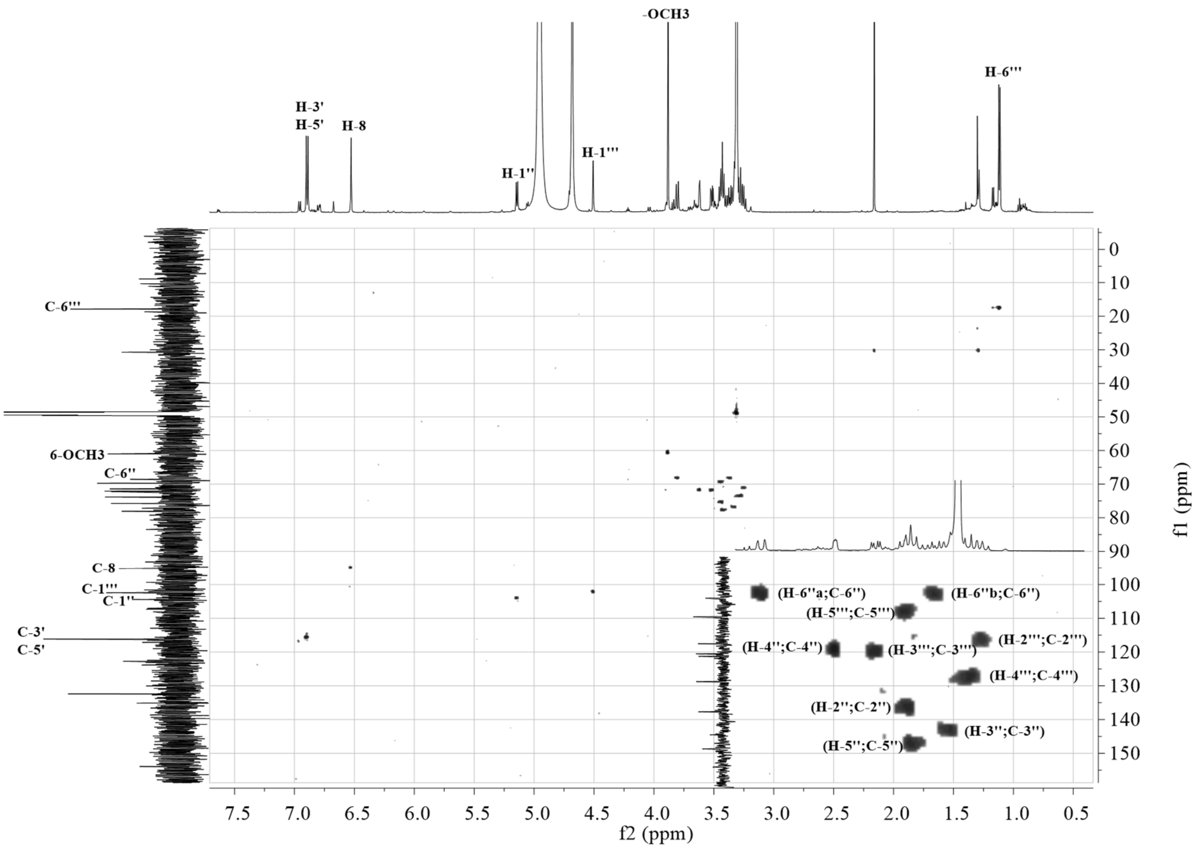

Figure S11. HSQC spectrum of compound **3** (CD_3OD, 600 MHz)

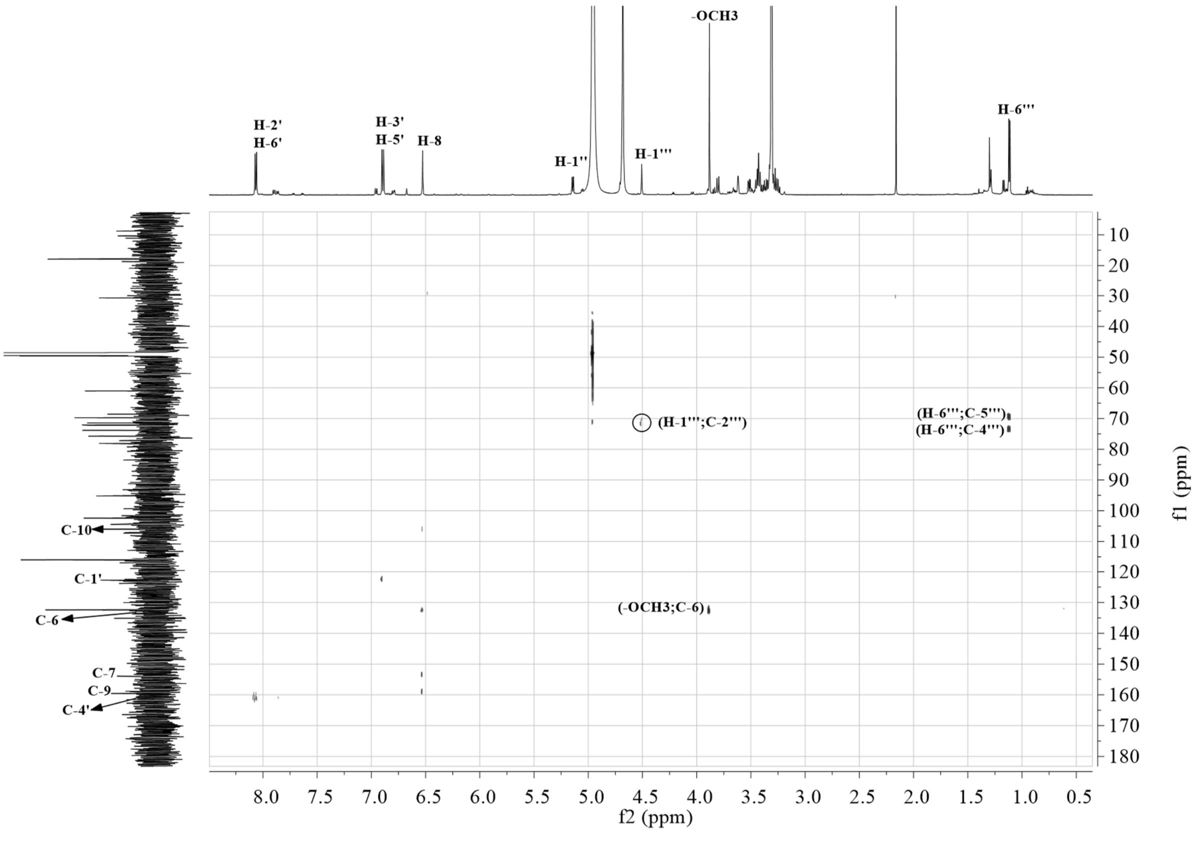

Figure S12. HMBC spectrum of compound **3** (CD_3OD, 600 MHz)

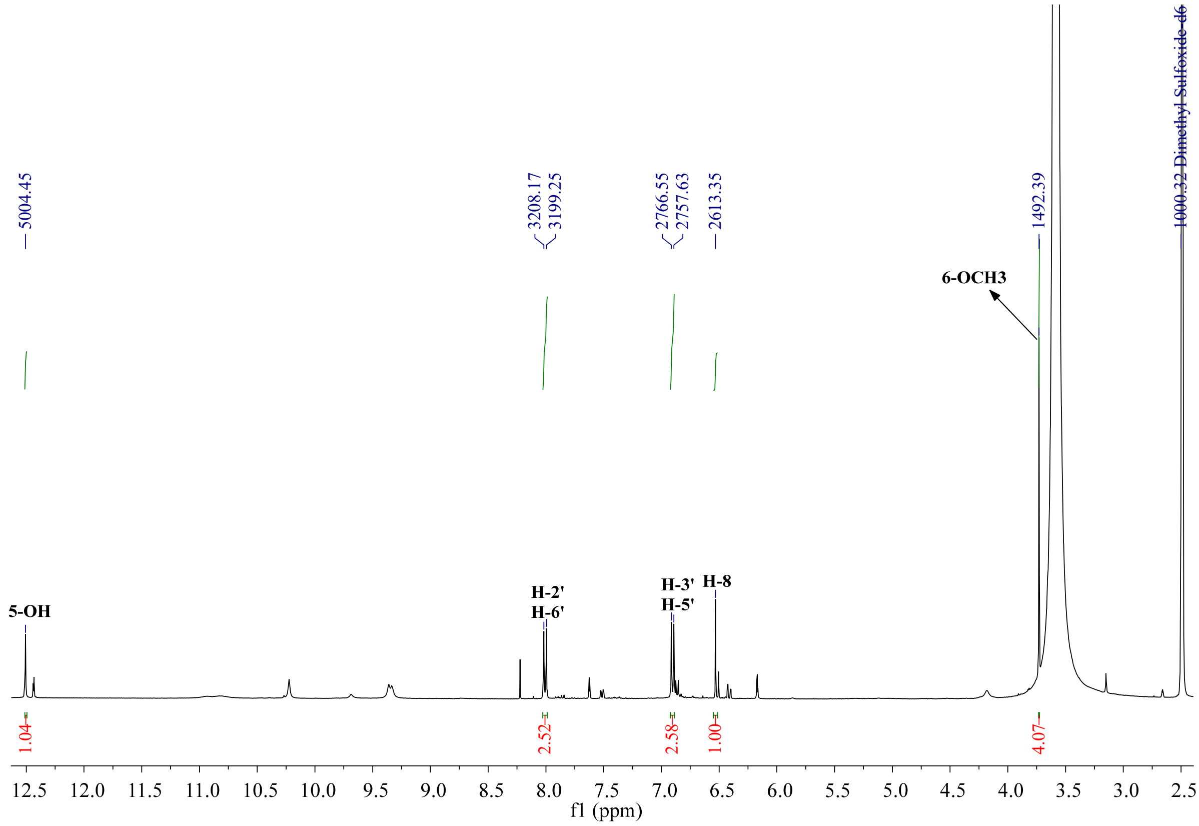

Figure S13. ^{1}H-NMR spectrum of compound **4** (DMSO-d_6, 400 MHz)

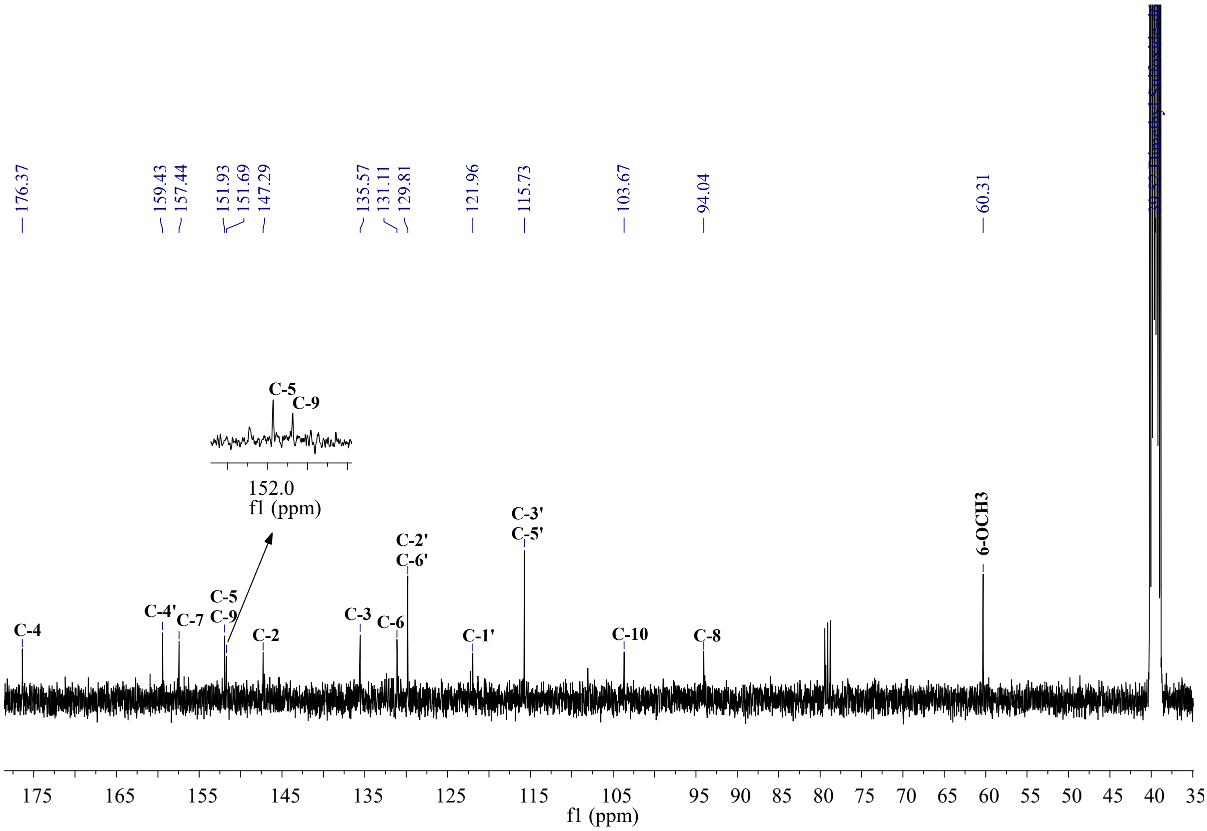

Figure S14. ^{13}C-NMR spectrum of compound **4** (DMSO-d_6, 400 MHz)

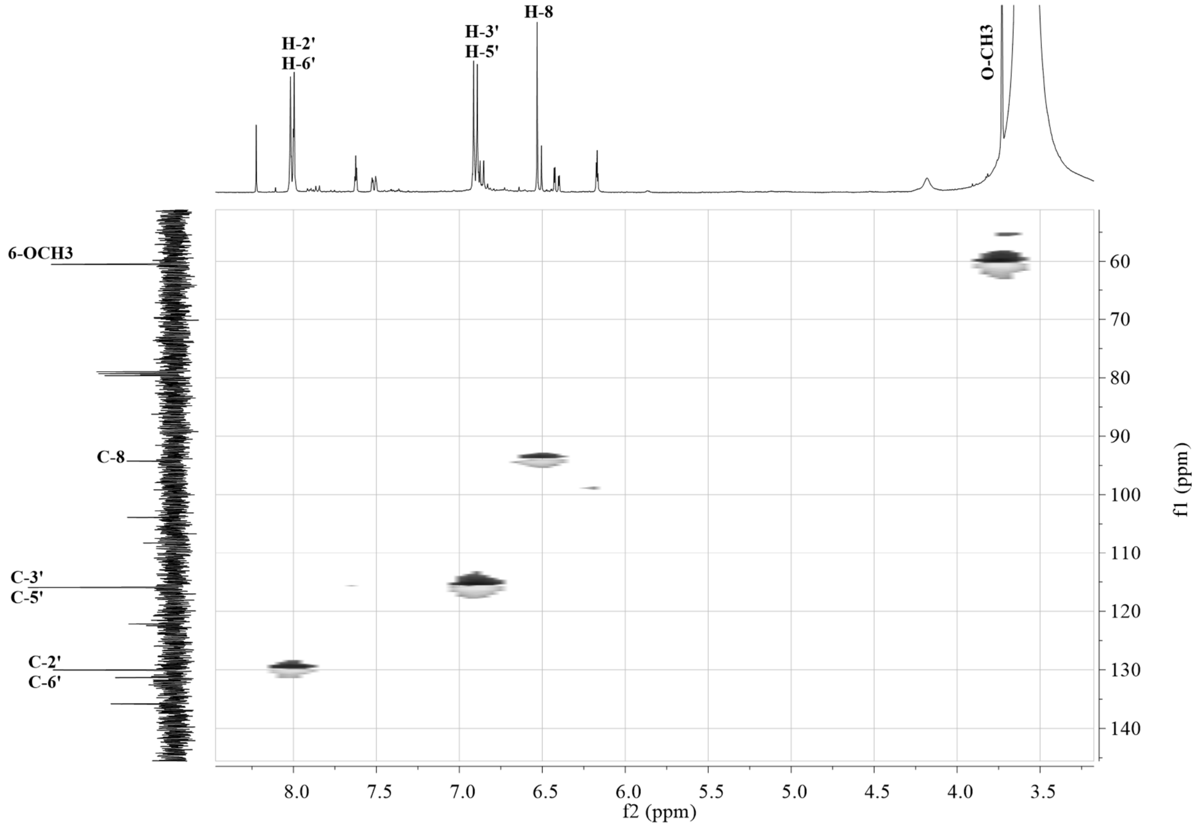

Figure S15. HSQC spectrum of compound **4** (DMSO-*d*$_6$, 400 MHz)

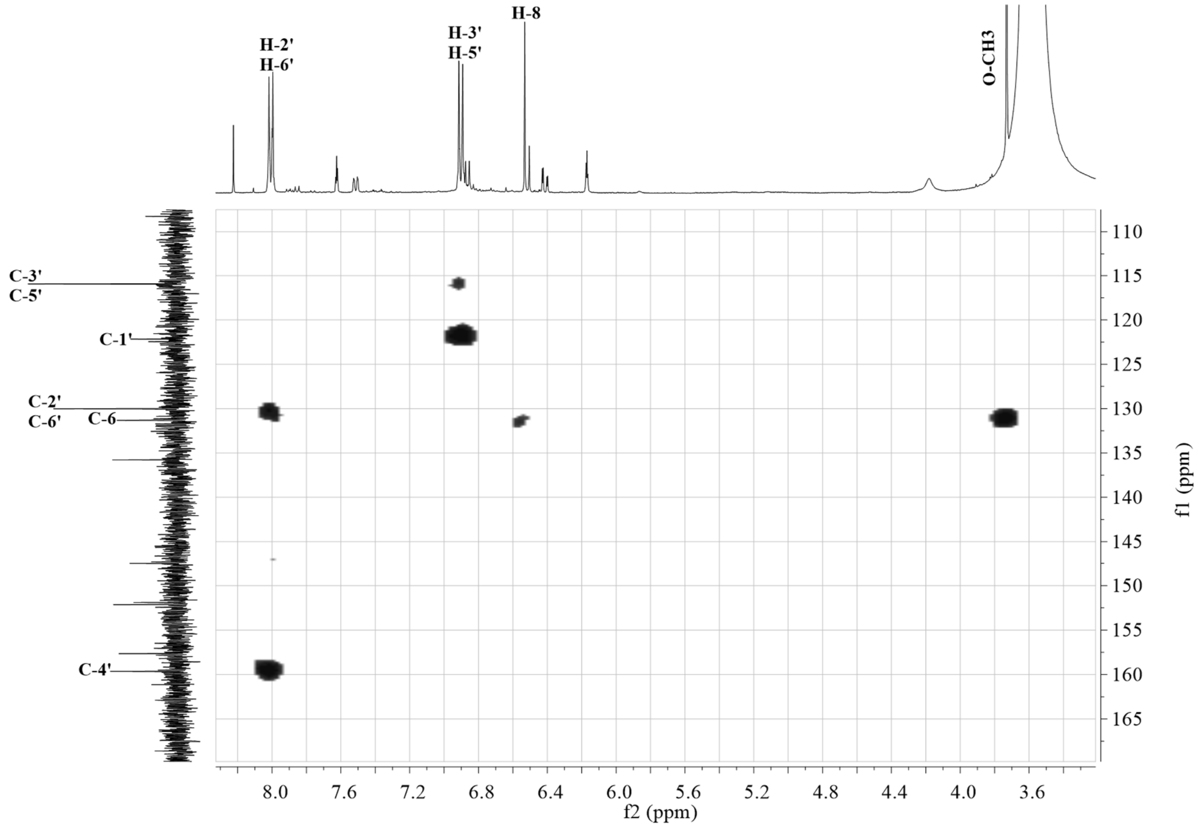

Figure S16. HMBC spectrum of compound **4** (DMSO-*d*$_6$, 400 MHz)

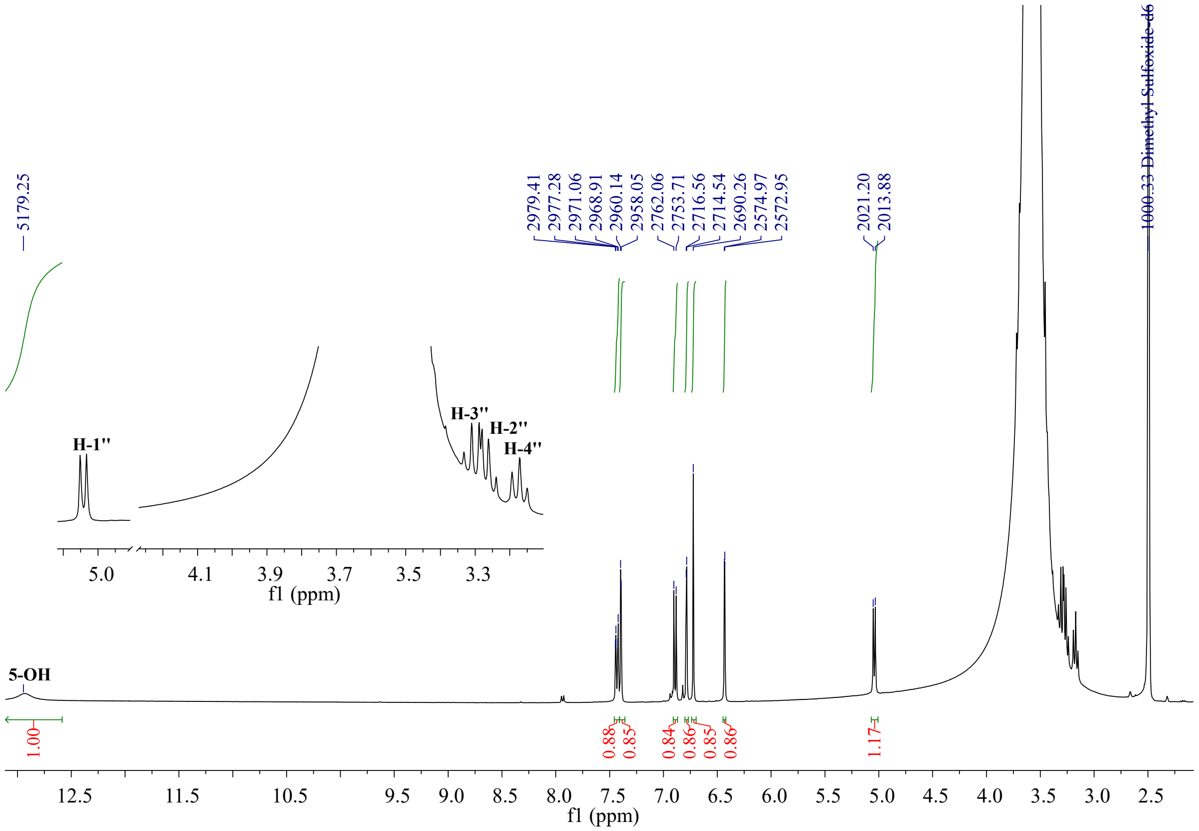

Figure S17. ^{1}H-NMR spectrum of compound **5** (DMSO-d_6, 400 MHz)

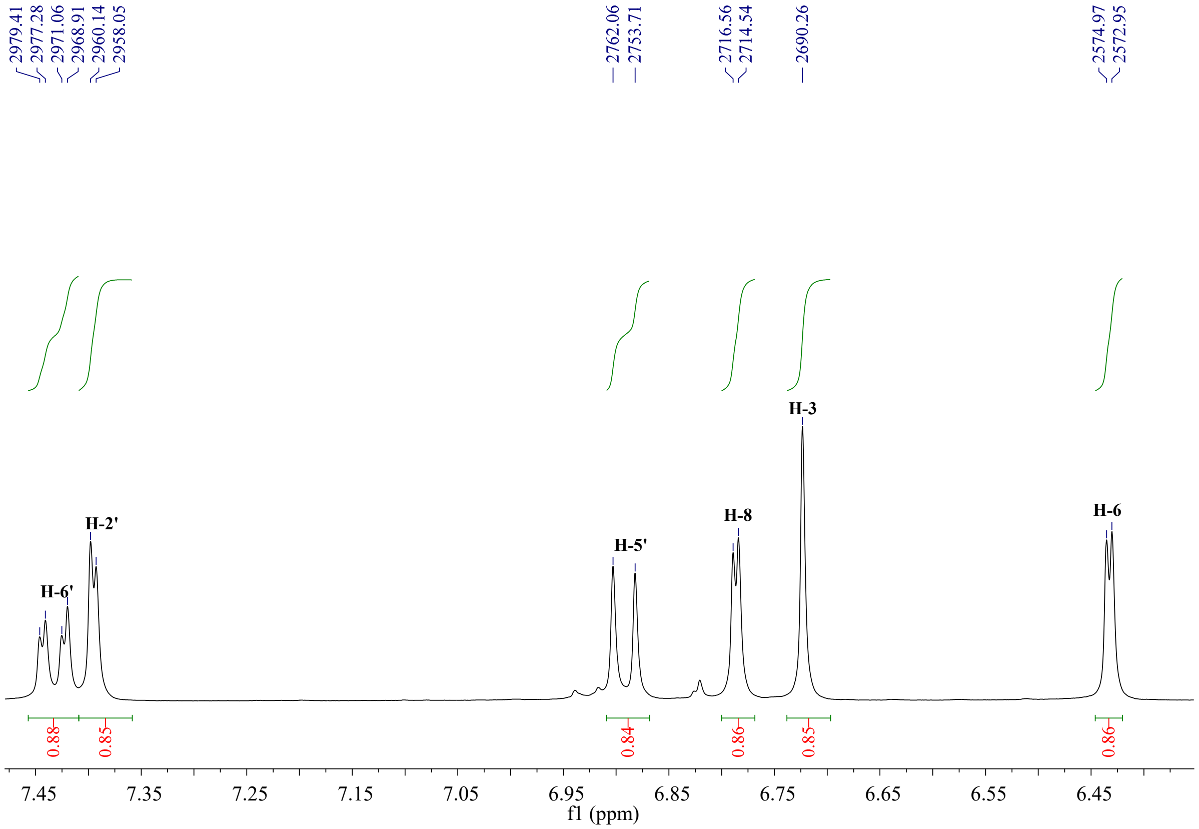

Figure S18. ^{1}H-NMR spectrum of compound **5** (DMSO-d_6, 400 MHz)

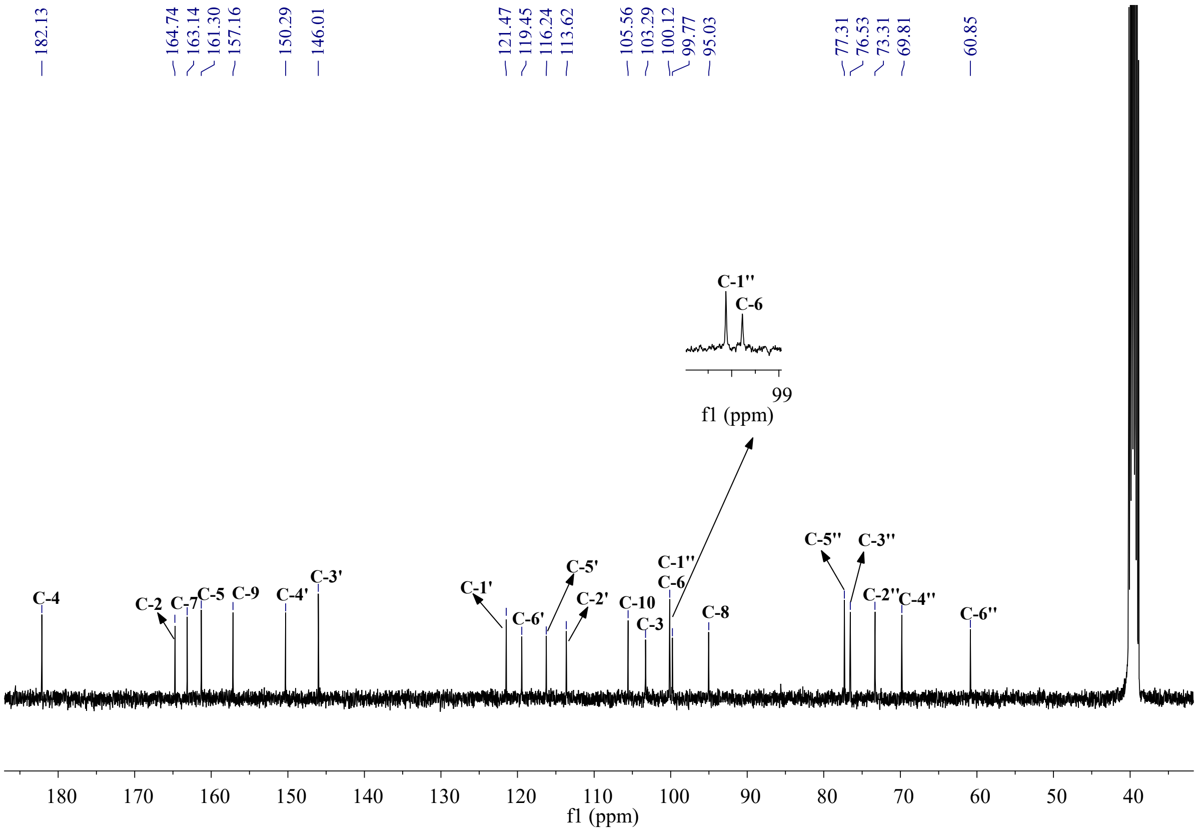

Figure S19. ^{13}C-NMR spectrum of compound **5** (DMSO-d_6, 400 MHz)

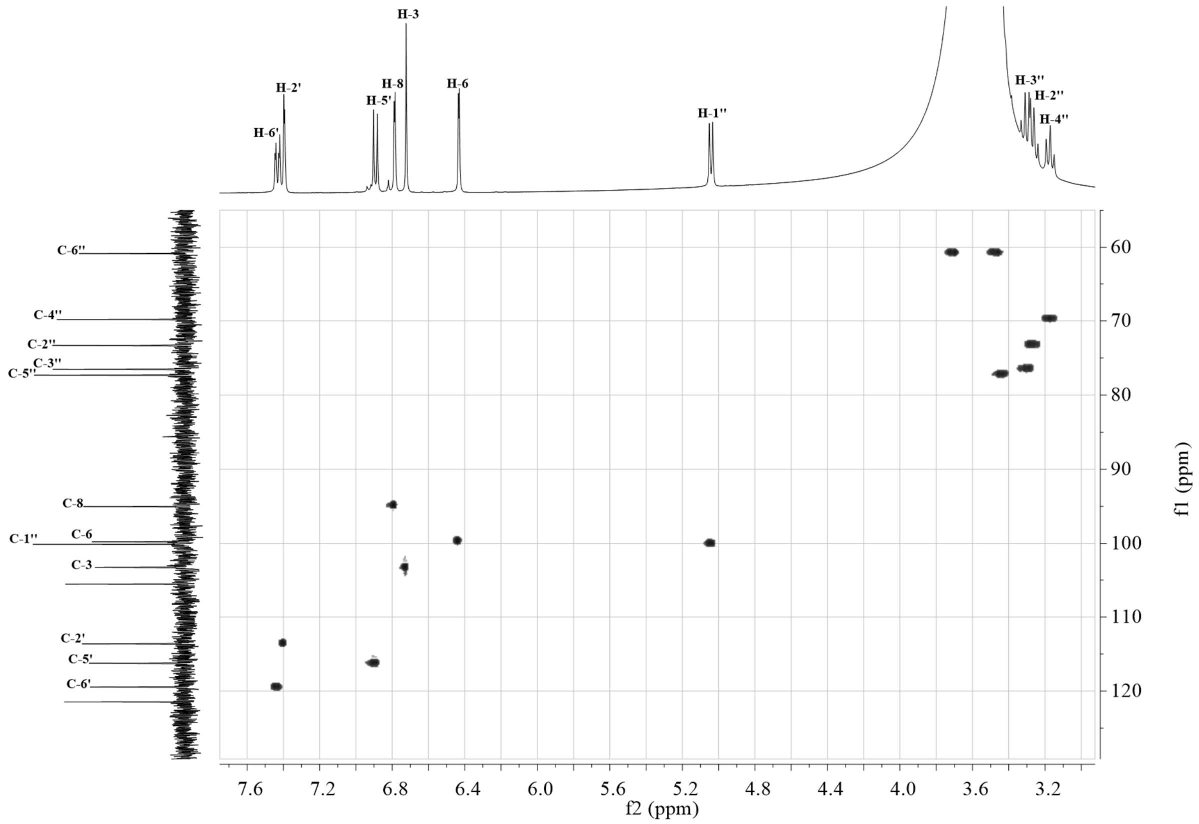

Figure S20. HSQC spectrum of compound **5** (DMSO-d_6, 400 MHz)

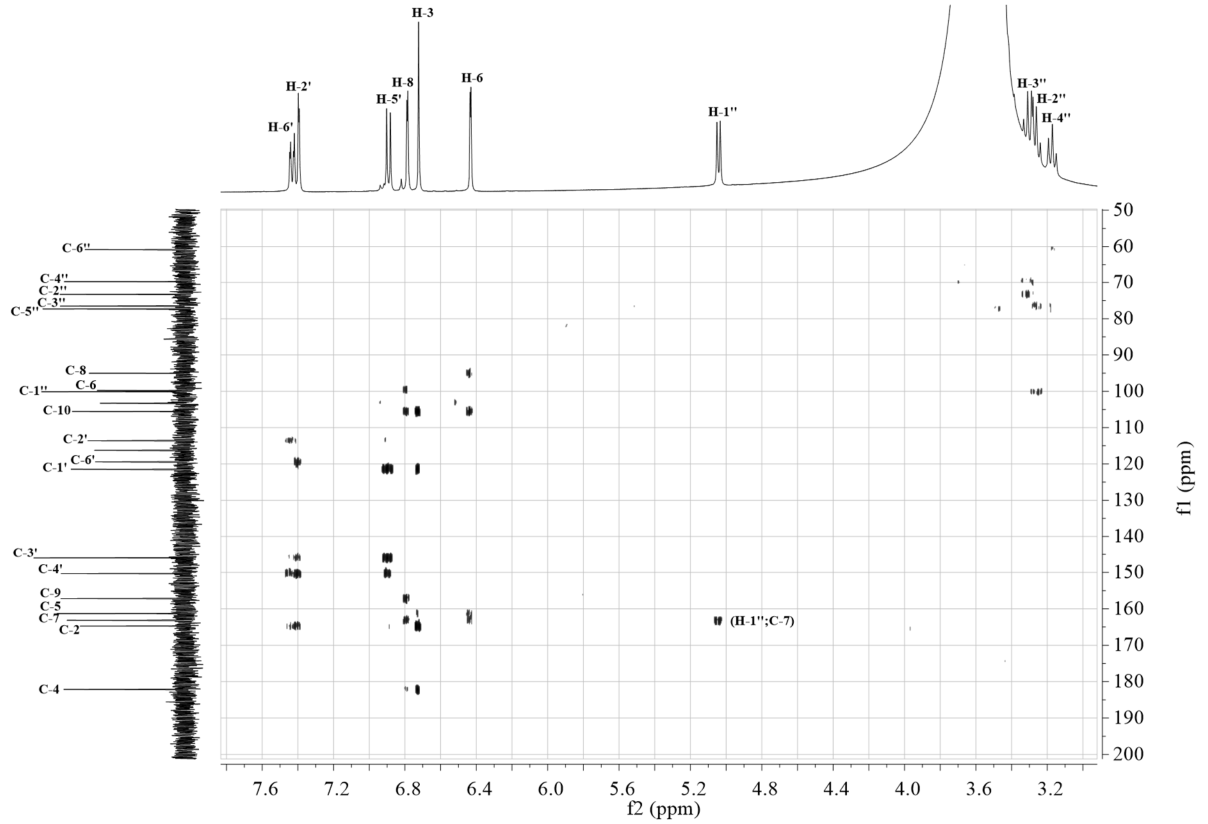

Figure S21. HMBC spectrum of compound **5** (DMSO-d_6, 400 MHz)

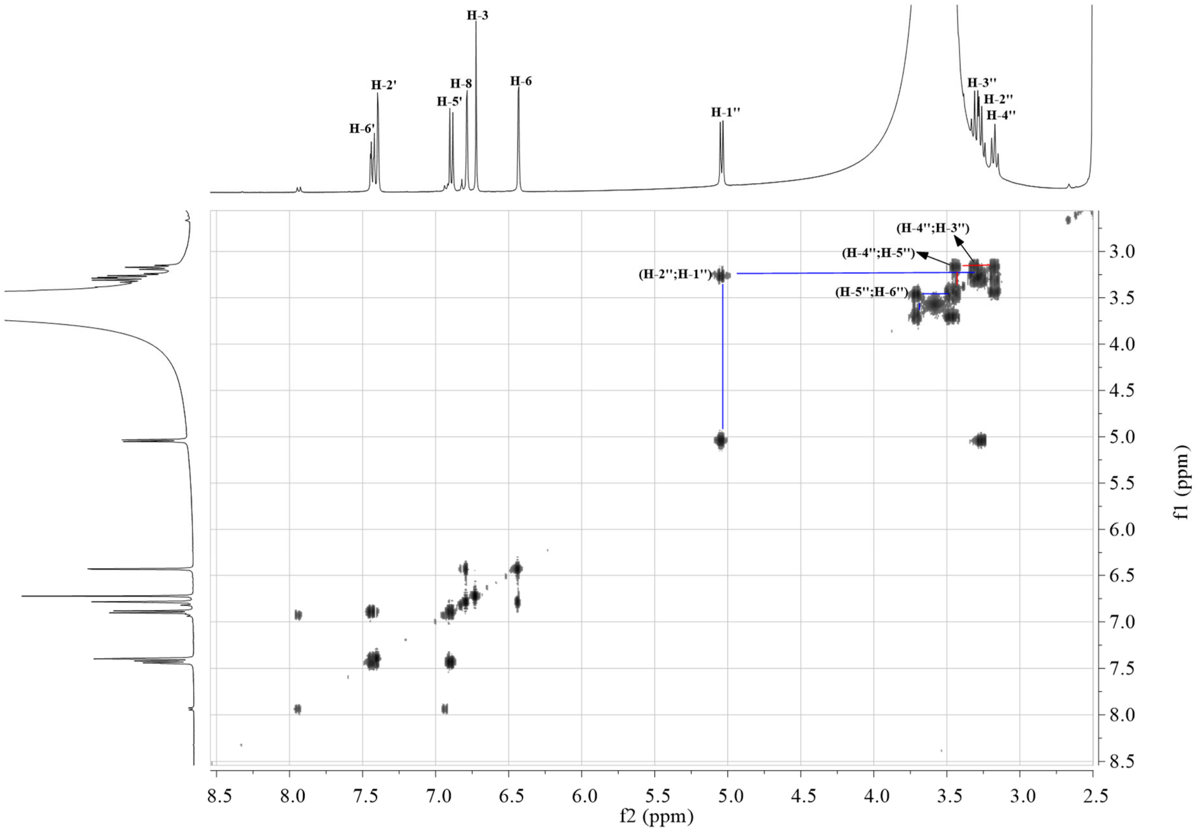

Figure S22. COSY spectrum of compound **5** (DMSO-d_6, 400 MHz)

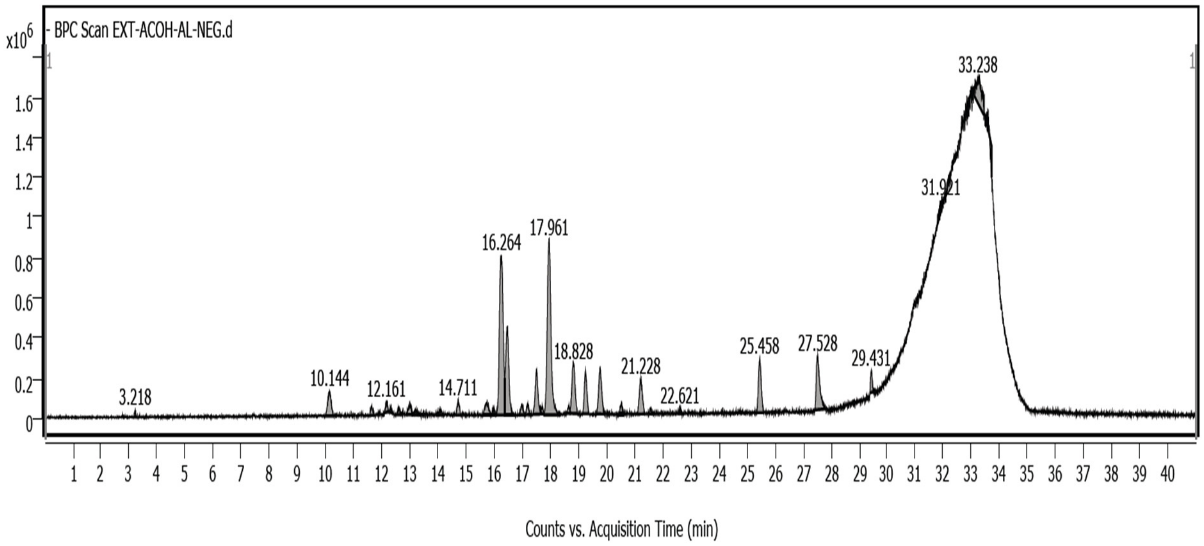

Figure S23. Base peak chromatogram of the AcOEt extract by HPLC-Q-TOF-MS in the negative ionization mode

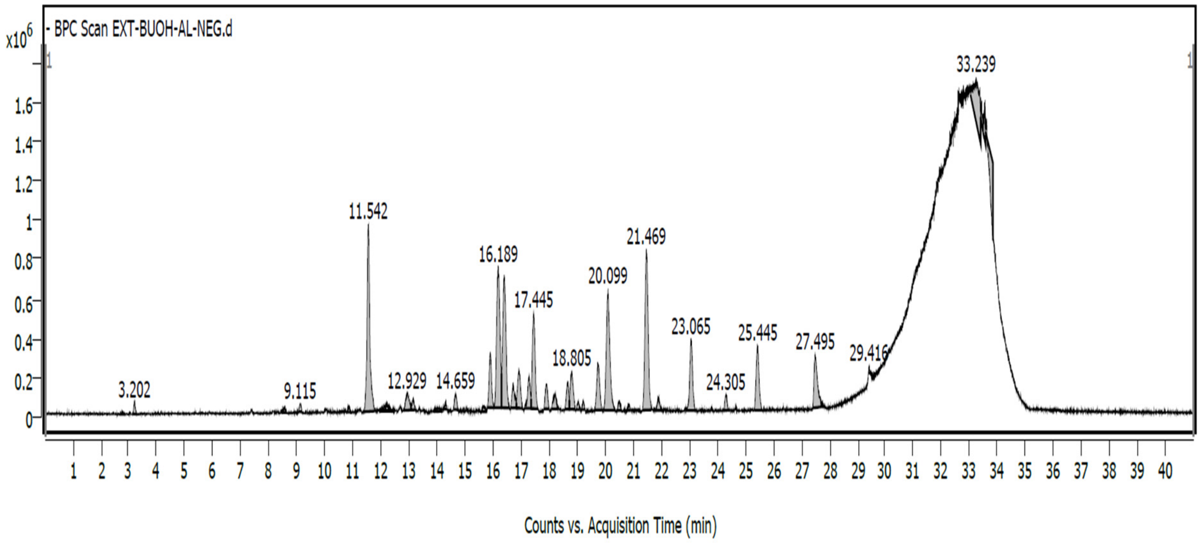

Figure S24. Base peak chromatogram of the BuOH extract by HPLC-Q-TOF-MS in the negative ionization mode
